# Dissecting cell membrane tension dynamics and its effect on Piezo1-mediated cellular mechanosensitivity using force-controlled nanopipettes

**DOI:** 10.1038/s41592-024-02277-8

**Published:** 2024-05-27

**Authors:** Ines Lüchtefeld, Igor V. Pivkin, Lucia Gardini, Elaheh Zare-Eelanjegh, Christoph Gäbelein, Stephan J. Ihle, Andreas M. Reichmuth, Marco Capitanio, Boris Martinac, Tomaso Zambelli, Massimo Vassalli

**Affiliations:** 1 Laboratory for Biosensors and Bioelectronics, ETH Zürich, Zurich, Switzerland; 2 Institute of Computing, Università della Svizzera Italiana, Lugano, Switzerland; 3 Swiss Institute of Bioinformatics, Lausanne, Switzerland; 4 National Institute of Optics, National Research Council, Florence, Italy; 5 European Laboratory for Non-Linear Spectroscopy, University of Florence, Florence, Italy; 6 Institute of Microbiology, ETH Zürich, Zurich, Switzerland; 7 Physics and Astronomy Department, University of Florence, Florence, Italy; 8 Victor Chang Cardiac Research Institute, Darlinghurst, New South Wales Australia; 9 James Watt School of Engineering, University of Glasgow, Glasgow, UK

**Keywords:** Membrane biophysics, Ion channels, Atomic force microscopy, Fluorescence imaging

## Abstract

The dynamics of cellular membrane tension and its role in mechanosensing, which is the ability of cells to respond to physical stimuli, remain incompletely understood, mainly due to the lack of appropriate tools. Here, we report a force-controlled nanopipette-based method that combines fluidic force microscopy with fluorescence imaging for precise manipulation of the cellular membrane tension while monitoring the impact on single-cell mechanosensitivity. The force-controlled nanopipette enables control of the indentation force imposed on the cell cortex as well as of the aspiration pressure applied to the plasma membrane. We show that this setup can be used to concurrently monitor the activation of Piezo1 mechanosensitive ion channels via calcium imaging. Moreover, the spatiotemporal behavior of the tension propagation is assessed with the fluorescent membrane tension probe Flipper-TR, and further dissected using molecular dynamics modeling. Finally, we demonstrate that aspiration and indentation act independently on the cellular mechanobiological machinery, that indentation induces a local pre-tension in the membrane, and that membrane tension stays confined by links to the cytoskeleton.

## Abstract (web summary)

FluidFM-based force-controlled nanopipettes enable control of mechanical stimuli for the investigation of Piezo1-induced mechanosensation in cell membranes.

## Main

Our cells need the ability to sense and react to the forces surrounding them in a multitude of contexts to ensure healthy development and avoid malfunctions^1^. While the importance of mechanical regulation in the development and physiology of tissues and organs has been extensively demonstrated^2^, the molecular and physical origin of this process has long remained elusive, mainly due to the lack of appropriate nanotechnology tools with which to study mechanosensitivity at the single-cell level. The relatively recent discovery of the major mechanically activated ion channel Piezo1 in 2010 (ref. ^3^) has opened up a new stream of investigation into mechanosensitive processes at the protein, cell and tissue level, the significance of which was highlighted by the 2021 Nobel Prize for Physiology^4^. It has been established that Piezo1 is activated by membrane tension^5^, which is defined as the force needed for the two-dimensional (2D) deformation of a unit length of membrane, and is driven by the disturbance of equilibrium distance between phospholipid molecules in the lipid bilayer^6^. Membrane tension is normally measured under the assumption of a mechanically homogeneous environment, with any stretch propagating across the whole membrane. While this is the case for liposomes and experiments performed in reconstituted environments, membranes of cells behave in a completely different way. The classical model of the cell membrane, with lipid rafts floating in a fluid environment, has recently been challenged^7^, suggesting a more complex structure characterized by the presence of immobile objects (transmembrane proteins anchored to the cytoskeleton) that create a network of dynamic fences hindering the propagation of mechanical stimuli across the membrane^8–10^ and resulting in a mechanically inhomogeneous environment^11^. Notably, when directly engaging the actin cortex, rapid long-range tension propagations can be observed^12^. This phenomenon of mechanical compartmentalization together with the clustering-enhanced response of Piezo1 (refs. ^13,14^) is deemed to allow cells to spatially resolve mechanical stimuli and enable directional responses as observed in durotaxis^15^ or endothelial shear sensing^16^.

The mechanical properties of the intracellular microenvironment, as determined by the interplay between the membrane and the cytoskeleton, are emerging as a key driver of cellular mechanosensation^17–19^, but a comprehensive method to study this mechanism is still missing. Patch-clamp electrophysiology is an invaluable tool to measure the ion currents associated with the opening of mechanosensitive ion channels^5,18,20,21^. While the aspiration pressure inside the patch pipette can be finely tuned, the indentation force applied on the contact region cannot be controlled, limiting the ability of this technique to modulate the local tensional state to the region inside the pipette^22^. Alternative approaches based on atomic force microscopy (AFM) and fluorescence microscopy have recently been suggested^21,23^. These methods integrate contact force sensitivity in the study of mechanosensitive ion channels and seem particularly suited to study the interaction processes between cells and with the extracellular matrix. However, they remain unsuitable to address the role of the mechanical intracellular microenvironment in Piezo1 activation. In fact, the presence of the micrometer-sized AFM probe displaces the ionic solution from the contact area, thereby compromising calcium imaging-based studies of local mechanosensitive ion channel activation.

Here, we present a novel method to study the single-cell mechanosensitive response based on force-controlled micropipettes, namely fluidic force microscopy (FluidFM)^24,25^. Using this unique tool, the membrane can be both indented with a well-defined force and at the same time aspirated inside the micropipette with a controlled pressure. By monitoring the Piezo1 response via calcium imaging and fluorescence microscopy, the contribution to the membrane tension induced by indentation as well as by aspiration can be decoupled and independently tuned. Moreover, we combined the platform with fluorescence lifetime imaging to take advantage of the recently developed fluorescent membrane tension probe Flipper-TR^26^. This integration enables us to finely resolve the spatial distribution and propagation of tension across the membrane upon controlled mechanical stimulation. Complemented by molecular dynamics simulations, this platform enables us to quantify the influence of different mechanical stimuli on the local membrane tension and the activation of mechanosensitive ion channels, and to confirm the local confinement of cellular membrane tension by the cytoskeleton.

## Results

### FluidFM with Ca^2+^ imaging measures mechanosensitivity

**Fig. 1:**
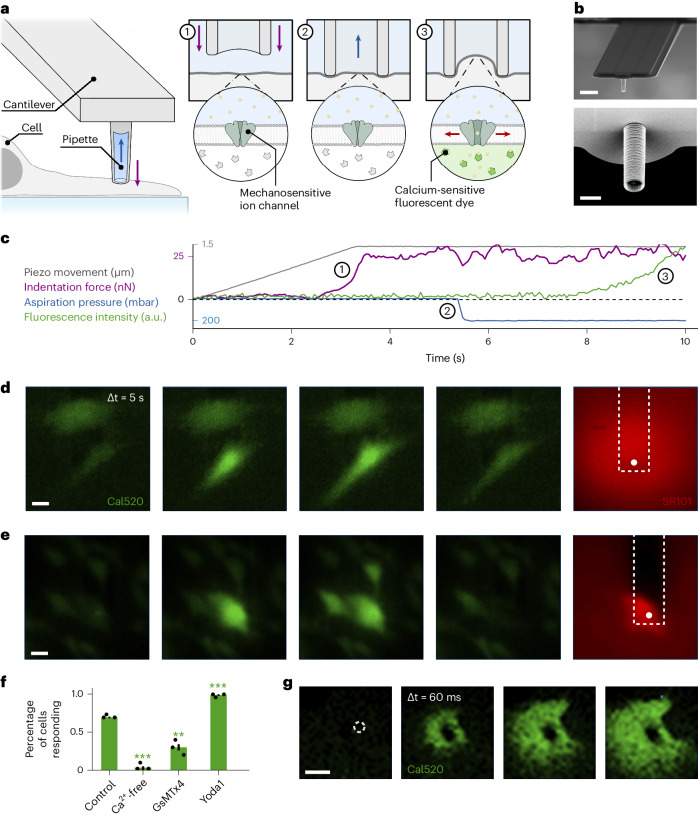
Measurement of mechanosensitivity combining FluidFM with Ca^2+^ imaging. **a**, Schematic diagram showing single-cell stimulation by (1) force-controlled indentation into the cell and (2) subsequent aspiration of the membrane, to (3) detect a calcium response by fluorescent imaging. **b**, Representative scanning electron microscopy (SEM) images of custom fabricated FluidFM probes with a cylindrical tip and a 2 µm inner diameter (scale bars: top, 10 µm; bottom, 2 µm). This imaging was repeated independently for each of the 32 FluidFM probes used in this study. **c**, Representative example protocol showing combined indentation and aspiration stimulus applied by FluidFM to adherent cells loaded with a calcium-sensitive dye. Numbers refer to the steps shown in **a**. **d**,**e**, Representative time series of a HFF cell stimulated with 25 nN indentation and 200 mbar aspiration (**d**), and an intentional rupture of an HFF cell membrane during stimulation with 25 nN indentation and 800 mbar aspiration (**e**). The position of the FluidFM cantilever and pipette tip are outlined by the white rectangle and circle, respectively. The green channel shows the cell-permeable calcium-sensitive dye Cal520-AM. The red channel shows the membrane-impermeable dye SR101, which is added to the pipette solution (scale bars, 20 µm). This imaging was repeated independently for each of the 320 cells for calcium imaging. **f**, Fraction of cells responding to 25 nN indentation and 200 mbar aspiration, in control conditions (*n* = 25), calcium-free solution (*n* = 10), after treatment with 500 µM of the mechanosensitive ion channel blocker GsMTx4 (*n* = 20), and with 10 µM of the Piezo1 activator Yoda1 (*n* = 25). Error bars correspond to 1 s.d. of three independent measurements with each of the number *n* of cells stated above. The control was significantly different to each of the other conditions, as determined using a two-sided *t*-test (*P* = 0.00005, 0.00267 and 0.00010, respectively). **g**, Representative high-resolution time series of initial calcium response to 100 nN indentation (scale bar, 5 µm). The high-resolution imaging was repeated independently for four cells. Source data

The experimental setup is based on a modified FluidFM device, optimized to achieve controlled nanoindentation and micropipette aspiration. FluidFM probes, used here as force-controlled micropipettes, are AFM cantilevers with an integrated microfluidic channel connected to a pressure controller that enables controlled fluid delivery or aspiration (Fig. 1a). Standard probes are available tipless or with a pyramidal tip, but these geometries are not suited for aspiration experiments. Therefore, we used customized probes with a cylindrical tip with an opening of 2 µm in diameter, designed to reproduce the geometry of glass capillaries (Fig. 1b)^27^. By coating the pipette with the recently developed copolymer PAcrAm-g-PMOXA with enhanced anti-fouling properties and stability, a combined indentation and aspiration protocol could be serially repeated up to 100 times without clogging the tip by cell debris^28,29^.

FluidFM stimulation was combined with calcium imaging by loading adherent cells with the cytosolic calcium sensor Cal520. Our setup enabled experiments to be carried out at physiological temperatures of 37 °C, which is not accessible by patch-clamp electrophysiology that is normally carried out at room temperature, and might therefore observe shifted protein and cytoskeletal kinetics. We chose fibroblasts as a model cell for their confirmed presence of Piezo1 (refs. ^14,30^).

### FluidFM stimulation yields Piezo1-mediated mechanosensation

As a first test of the proposed stimulation and readout method, cells were subjected to a combined stimulus of 25 nN indentation force and 200 mbar (≙150 mmHg) aspiration pressure (Fig. 1c): Indeed, cells showed an increase in fluorescence signal related to a rise in calcium concentration throughout the whole cell (Fig. 1d and Supplementary Video 1). As a control to confirm that this cell-wide calcium rise is not associated with any unwanted membrane rupture during the pipette–membrane contact, the red hydrophobic dye Sulforhodamine 101 (SR101) was added to the solution inside the FluidFM microchannel. Given that the dye did not enter the cell, we concluded that the membrane remained intact during the mechanical stimulus. To remove any doubt, we intentionally ruptured the cell membrane by applying an 800 mbar (≙600 mmHg) aspiration pressure and immediately noted SR101 entering the damaged cell (Fig. 1e and Supplementary Video 2). In these cases, not only the damaged cell but also the surrounding ones showed an increase in calcium concentration, probably due to ATP release caused by membrane rupture and consequent activation of purinergic receptors^31^.

To better elucidate the nature of the mechanically induced calcium increase, we repeated the experiment in different conditions, noting how many cells showed at least a threefold fluorescence increase above baseline (Fig. 1f). In the control case, with calcium-containing physiological solution in both the bath and pipette, 70% of the cells responded to the stimulus. When replacing both the bath and pipette solution with EGTA-containing calcium-free solution, only 3% of the cells responded, suggesting a mechanism reliant on extracellular calcium, such as the activation of mechanosensitive ion channels. To confirm this, upon treatment with the mechanosensitive ion channel blocker GsMTx4 (ref. ^32^) only 25% of the cells showed a cell-wide calcium increase. This points towards the involvement of mechanosensitive ion channels, known to show reduced, but not completely abolished activity after GsMTx4 treatment^33^. By contrast, treatment with the Piezo1-specific activator Yoda1 (ref. ^34^) increased the likelihood of cell response to 95%. From this experiment we could deduce that the mechanosensitive ion channel Piezo1 plays a key role in the observed response in fibroblasts (Fig. 1f).

Furthermore, we investigated the spatial origin of the calcium response by coupling the FluidFM stimulation setup to a fast confocal laser scanning microscope. A representative calcium response is shown in Fig. 1g: the calcium rise clearly originates at the stimulation site and spreads throughout the whole cell with a speed of 22.4 ± 4.8 µm s^−1^, which is in agreement with the previously measured diffusion speed of calcium in the cytosol of 10–50 µm s^−1^ (ref. ^35^).

### Flipper-TR imaging during FluidFM reveals tension changes

**Fig. 2:**
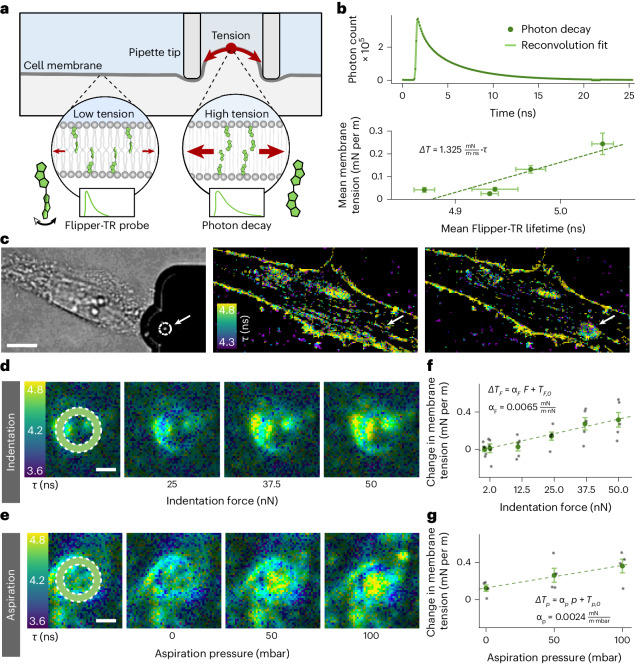
Flipper-TR imaging during FluidFM stimulation quantifies tension changes. **a**, Schematic diagram of fluorescent measurement of membrane tension changes during FluidFM stimulation by monitoring the fluorescence lifetime of Flipper-TR with FLIM microscopy. **b**, Top: representative time distribution of single photon counts after excitation for Flipper-TR, and the 2-exponential reconvolution fit to obtain the fluorescence lifetimes. Photon counts were measured independently for all 42 FLIM measurements. Bottom: average Flipper-TR lifetimes (*n* = 21, 22, 22, 23 and 23 cells for the osmotic conditions of 100, 150, 200, 250 and 300 mOsm kg^−1^, respectively) corresponding to membrane tension values measured by optical tweezer tether pulling (*n* = 7, 7, 8, 8 and 10 cells per osmotic condition) on HFF-1 cells under varying osmotic pressure. Error bars correspond to 1 s.e. Linear fit with *R*^*2*^ = 0.890. **c**, Representative bright field (left), and fluorescence lifetime images of a cell loaded with Flipper-TR before (middle) and after (right) indentation with a 25 nN force with the FluidFM pipette (white circle and arrow) (scale bar, 5 µm). Dark pixels are not part of the membrane cross-section and therefore do not emit sufficient photons to obtain significant FLIM statistics. This imaging was repeated independently for 92 cells shown in Supplementary Information 7. **d**,**e**, Representative time series of fluorescence lifetime imaging of the FluidFM pipette on one Flipper-TR loaded cell during increasing indentation (**d**) and aspiration (**e**) (scale bars, 1 µm). White circles in the first image show the position of the wall of the pipette tip. This imaging for indentation and aspiration was repeated independently for 5 and 4 cells, respectively. **f**,**g**, Change in membrane tension with increasing indentation (**f**) and aspiration (**g**), averaged from independent measurements on 5 and 4 cells, respectively. Aspiration is applied while cells are indented at 25 nN. Error bars correspond to 1 s.e. Linear fits with *R*^*2*^_*F*_ = 0.979 and *R*^*2*^_*P*_ = 0.996, respectively. Source data

The previous results suggest that Piezo1 channels are activated only locally. Given that Piezo1 activation has been associated with changes in membrane tension^5,18,36^, we aimed to quantify the extent and propagation of membrane tension changes upon mechanical stimulation by FluidFM. For this, we combined the FluidFM system with a fluorescence lifetime imaging microscopy (FLIM) setup to exploit the recently developed fluorescent membrane tension probe Flipper-TR (ref. ^26^) (schematic shown in Fig. 2a). This fluorophore is inserted into the lipid membrane where its flippers rotate depending on the tension and lipid environment of the surrounding membrane, thereby altering its fluorescence lifetime. Thus, imaging the lifetime of this fluorescent probe can provide quantitative insights into the magnitude of membrane tension changes and enable the tracking of its spatial distribution over time. This approach offers a spatially resolved measure of the local tension, which is normally not accessible in micropipette aspiration or tether pulling experiments in which the local average membrane tension is measured. To date, Flipper-TR has been used to study spatial membrane tension changes in a static environment^37–39^. Here, we went a step further by combining Flipper-TR spatial membrane tension imaging with dynamic mechanical stimulation and additional extraction of real-time information on tension propagation.

To perform FLIM imaging on Flipper-TR-incubated cells in the presence of the FluidFM tool, we modified the AFM stage and scan head as documented in the Methods and Supplementary Information 1. To obtain a statistically relevant value for the fluorescence lifetime, a minimum of 1,000 photons per pixel must be collected to reliably perform a 2-exponential reconvolution fit of the time distribution of single photon counts upon excitation (Fig. 2b, top)^40,41^. The larger component of the Flipper-TR lifetime extracted from the fit is directly related to the membrane tension. Once established, the spatial distribution of membrane tension can be directly observed in the FLIM images during mechanical stimulation. Figure 2c shows a reference experiment in which the FluidFM probe is indenting into the cell with a 25 nN force. The colors, from blue to yellow, encode for the larger fluorescence lifetime component *τ*, which is directly proportional to the membrane tension *T*. The increased tension induced locally by the FluidFM tip is clearly visible in the images. To convert the measured Flipper-TR lifetime changes to membrane tension changes, a calibration was performed on HFF-1 (human foreskin fibroblast) cells by optical trap tether pulling during hypoosmotic shock, yielding a conversion factor of 1.325 ± 0.392 (mN m^−1^) ns^−1^ (Fig. 2b, bottom, and Supplementary Information 2). Additional measurements with the fluorescent lipid order probe, Laurdan, confirmed that here, Flipper-TR is indeed an indicator of membrane tension changes and not of lipid order (Supplementary Information 3).

### Indentation and aspiration locally increase tension

We further analyzed the behavior of the membrane tension during FluidFM stimulation, combining indentation and aspiration. Focusing on the stimulation site (Fig. 2d–g), the Flipper-TR lifetime appears clearly altered where the membrane is in contact with the ring-shaped edge of the hollow cylindrical FluidFM probe, and the local tension changes can be monitored as a function of the ongoing mechanical stimulus. With increasing indentation from 2 to 50 nN, an increasing lifetime was observed (Fig. 2d). Similarly, when applying an increasing aspiration stimulus from 0 to 100 mbar (≙75 mmHg) on a cell indented with a constant 25 nN force, an overall lifetime increase was observed (Fig. 2e).

In Fig. 2f,g the Flipper-TR lifetime changes were measured on 4 and 5 cells during increasing indentation and aspiration^42^, respectively, and converted into membrane tension changes using the calibrated conversion factor from Fig. 2b. Both indentation and aspiration induce a linear increase of the local membrane tension, with total changes of 0.31 mN m^−1^ and 0.24 mN m^−1^, respectively. The coefficients of the linear fit of the tension change *α*_*F*_ = 0.0065 (mN m^−1^) nN^−1^ and *α*_*p*_ = 0.0024 (mN m^−1^) mbar^−1^ can be used to compare their net effect.

### Combined stimuli affect the number of activated Piezo1 channels

**Fig. 3:**
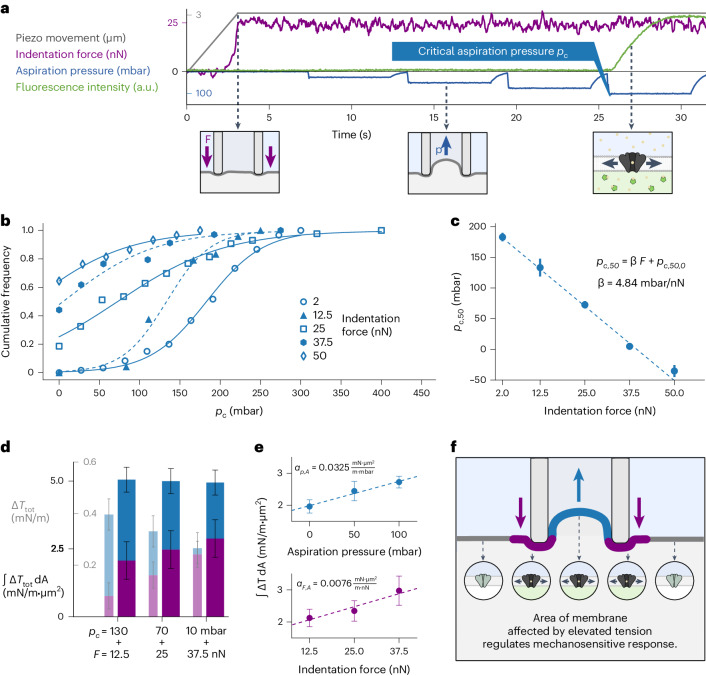
Effect of combined mechanical stimuli on Piezo1 channel activation. **a**, Representative protocol of combined indentation and aspiration to determine the critical aspiration pressure as a function of the indentation force. This protocol was repeated independently for all 399 combined indentation and aspiration measurements included in this study. **b**, Cumulative frequency of critical aspiration pressures *p*_*C*_ measured for each indentation force, fitted with a sigmoid function to determine *p*_*C,50*_ as the pressure of half-maximum response, measured in three independent repeats with a total of 60, 24, 43, 34 and 59 cells for the force steps of 2, 12.5, 25, 37.5 and 50 nN, respectively. **c**, Half-maximum critical aspiration pressure *p*_*C,50*_ (as determined from **b**) as a function of the indentation force *F*. Linear fit with *R*^*2*^ = 0.997. The error bars correspond to the standard deviation of the sigmoid fit plus a measure for the goodness of fit of the sigmoidal model, as defined in the Methods section. **d**, Total membrane tension change *ΔT*_tot_ (light bars) integrated over the corresponding affected area ʃΔ*T*_tot_ d*A* (dark bars) induced by indentation and aspiration, calculated as *ΔT*_tot_ $=$ *α*_*F*_*∙F* + α_*p*_*∙p* and $\int \Delta {T}_{{{\mathrm{tot}}}}{\rm{dA}}={\alpha }_{F,A}\cdot F+{\alpha }_{p,A}\cdot p$ from the *α* values measured in Fig. 2f,g and Fig. 3e, respectively. Error bars correspond to 1 s.d. of the linear fit parameters determined from Fig. 2f,g and Fig. 3e. **e**, Mean tension change Δ*T* integrated over membrane area ʃΔ*T* d*A* for increasing indentation (bottom) and aspiration (top), averaged from independent measurements on 5 and 4 cells, respectively. Error bars correspond to 1 s.e. Linear fits with *R*^*2*^_*F*_ = 0.963 and *R*^*2*^_*P*_ = 0.989, respectively. **f**, Schematic diagram of the role of the area and number of channels affected during a stimulus by combined indentation and aspiration. Source data

In standard micropipette aspiration and patch-clamp experiments, the force exerted by the pipette tip on the membrane at contact is not controlled. Although the applied contact force is typically held minimal, the indentation with the pipette does induce a local increase in membrane tension, as shown by the Flipper-TR measurements in Fig. 2. The FluidFM force-controlled micropipette enables us to independently control local indentation force and aspiration pressure, and thereby to assess their cooperative effect on mechanosensitive ion channels via calcium imaging. We defined an experimental protocol in which the probe is first brought into contact with the cell membrane at a controlled indentation force *F* (Fig. 3a). Subsequently, aspiration pressure pulses of increasing intensity are applied while monitoring the fluorescence level as a sign of the elicited mechanosensitive calcium response. Between pressure pulses, the aspiration was paused for 1 s to ensure equilibration of Piezo1 and to minimize the impact of channel inactivation^43^. This protocol was serially executed on several cells, with the application of a positive pressure pulse after each unloading of the cantilever, to detach the membrane and clean possible cell debris from the pipette. While repeating the measurement with indentation forces between 2 nN and 50 nN on ≥60 cells each (see Supplementary Information 4 and Supplementary Video 3 for measurements at higher indentation forces), the critical aspiration pressure *p*_*C*_ inducing the calcium response is recorded for each cell, achieving a throughput of 1–2 cells per minute. For each indentation force, the cumulative histogram of the events is constructed, representing the activation probability for the specific condition (Fig. 3b).

Fitting the data for each condition with a Boltzmann curve determines the half-maximum critical aspiration pressure *p*_*C,50*_ as the pressure at which 50% of the cells respond. Considering the dependence of *p*_*C,50*_ on the average indentation depth (Supplementary Information 5), a decreasing, but non-linear relationship is seen. This result suggests that Piezo1 response and/or membrane tension are affected not only by the geometrical stretch of membrane area but also by the presence of the underlying cytoskeleton. However, examination of the *p*_*C,50*_ as a function of the indentation force *F* (Fig. 3c) shows that the critical aspiration pressure decreases for increasing indentation force, exhibiting a clearly linear trend with a slope of β = 4.84 ± 0.05 mbar nN^−1^. The linearity of the *p*_*C,50*_(*F*) curve provides a reliable readout to study changes in the mechanosensitive response of a cell population (as detailed later in Fig. 5a,b). This force-dependent readout is enabled only by a force-controlled indenter, showing the unique suitability of the FluidFM for this method. The maximum stimuli of 50 nN indentation and 181 mbar (≙136 mmHg) aspiration required to elicit a mechanosensitive response with this method are comparable to values previously reported in the literature, as discussed in more detail in Supplementary Information 6.

The linear decay of *p*_*C,50*_(*F*) hints at a mechanism whereby colocalized indentation and aspiration stimuli add together to induce a critical total membrane tension, leading to the activation of mechanosensitive ion channels. To understand the nature of this cooperative effect, we can refer back to the Flipper-TR measurements in Fig. 2f,g and use the measured coefficients *α*_*F*_ and *α*_*P*_ to convert the applied indentation force *F* and aspiration pressure *p* into the corresponding average membrane tension changes *ΔT*_*F*_ = *α*_*F*_*·F* and *ΔT*_*P*_ = *α*_*P*_*·p*. Summing the average tension increase from application of indentation and aspiration for the combinations determined in *p*_*C,50*_(*F*) in Fig. 3c, it can be seen that the total membrane tension change *ΔT*_tot_ leading to a mechanosensitive calcium response is not constant (Fig. 3d, light bars). If we assume that neither indentation nor aspiration change the sensitivity and conductivity of each Piezo1 channel, this result highlights that the cell-wide calcium response does not depend solely on the average membrane tension increase induced at the pipette.

In fact, the observed cell-wide calcium response is not elicited by a single, but by a number of Piezo1 channels, and the likelihood of each channel being activated is dependent on the local membrane tension surrounding it^5^. Piezo1 is known to be very mobile across the plasma membrane, exploring different microdomains^30^ where clustering mostly occurs at adhesion sites^14^. In the work presented here, we challenged the apical membrane with a pipette aperture far larger than the average size of Piezo1 clusters^19^. For this reason, we can assume an equal number of channels in each portion of membrane area, and therefore estimate a measure for the number of channels activated during a stimulus by integrating the local membrane tension changes over all portions of membrane area at the stimulation site. For this, we used the Flipper-TR measurements in Fig. 2d–g and summed the lifetime of all pixels that show a significant increase during mechanical stimulation, thereby obtaining the integrated membrane tension change over the affected membrane area ʃΔ*T* d*A* for different stimulation intensities by indentation and aspiration (Fig. 3e). Using this approach, we recalculate the relevant conversion coefficients as *α*_*F,A*_ = 0.0076 (mN m^−1^)·(µm^2^ nN^−1^) and *α*_*p,A*_ = 0.0325 (mN m^−1^)·(µm^2^ mbar^−1^) for indentation and aspiration, respectively. These coefficients are used to recalculate the bar plot (Fig. 3d, dark bars), now showing that the effects of both indentation and aspiration independently contribute to the mechanosensitive calcium response by activating the same mechanism, ultimately associated with the tension increase in the local microenvironment and the number of Piezo1 mechanosensitive ion channels affected by it (Fig. 3f).

### Tension changes do not propagate across the cell membrane

**Fig. 4:**
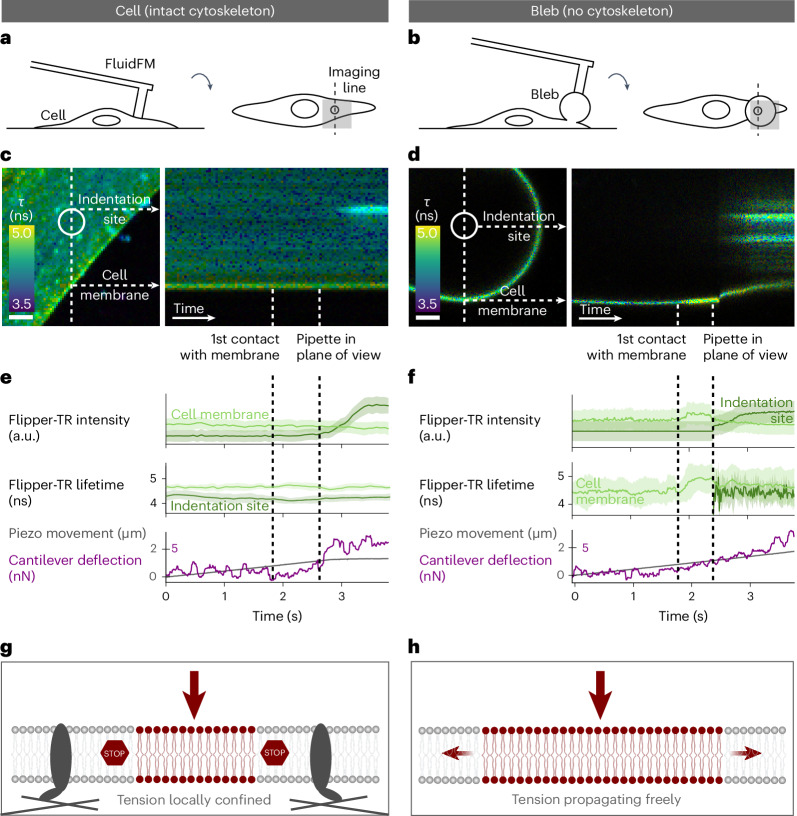
Propagation of tension changes throughout the intact cell and detached membrane. **a**,**b**, Schematic diagrams of the stimulation and imaging setup for an intact cell (**a**) and a cell-attached membrane bleb (**b**), as seen from the side (left) and bottom (right). **c**,**d**, Kymographs for real-time fluorescence lifetime imaging of Flipper-TR on the cross-section of an intact cell (**c**) and a membrane bleb (**d**), during indentation with 7.5 nN (scale bars, 2 µm; timescale: **c**, 80 ms px^−1^; **d**, 20 ms px^−1^). **e**,**f**, Timelines of the AFM quantities piezo movement and cantilever deflection (bottom), and the FLIM quantities average Flipper-TR intensity and average fluorescence lifetime (top) over all pixels at the indentation site (dark green) and on the outer membrane (light green). The shaded areas correspond to one standard deviation of the Flipper-TR intensities and lifetimes. The vertical black dashed lines represent the timepoints of the first contact of the pipette with the membrane, and the appearance of the pipette in the imaging plane, respectively. **g**,**h**, Schematic models for the propagation of membrane tension in an intact cell membrane (**g**) and the membrane of a cytoskeleton-free bleb (**h**). Source data

Interestingly, when using the combination of FluidFM with Flipper-TR imaging presented in Fig. 2 to examine the lifetime at the spatial scale of whole cells, no significant changes could be observed during FluidFM stimulation, neither for strong indentation nor aspiration (Supplementary Information 7). Therefore, the question remains as to whether fast transient tension changes might occur on the cell-wide level and are averaged out during the typical imaging time of 30 s necessary to obtain a significant FLIM photon count for a full cell frame. Therefore, it is necessary to collect, on one hand, the statistically required 1,000 photons per pixel, but on the other hand to achieve a minimal frame rate in the millisecond regime. This was accomplished by performing imaging not as a 2D image, but along a cross-sectional line across the cell membrane and the stimulation site (Fig. 4a,b). Assembling the imaged lines next to each other, a tension kymograph is created, which provides information not only on the spatial, but also on the temporal distribution of membrane tension with a 20–80 ms time resolution (Fig. 4c,d).

A reference experiment on a cell is shown in Fig. 4c. When indenting with a 7.5 nN force, the FluidFM probe comes into contact with the cell membrane after 1.8 s (note the rise of the AFM cantilever deflection in Fig. 4e). At this timepoint neither the fluorescence intensity nor the Flipper-TR lifetime at the cell membrane or indentation site show any significant changes within the time resolution of 80 ms. After 2.6 s, the FluidFM probe enters the plane of view and the intensity at the indentation site increases significantly. The lifetime at the indentation site shows a slight increase, while no significant changes are observable at the cell membrane. In experiments repeated on five separate cells with an indentation of 50 nN (ref. ^42^), no changes in Flipper-TR lifetime in the cell membrane are observed (Supplementary Information 8a). This confirms the previous observation of the localized nature of membrane tension changes in cells, which is consistent with the anchored transmembrane fence model, recently demonstrated using single-cell tether pulling experiments^8,9^.

The experiment was repeated on cell membrane blebs, which are known to be devoid of the actin cytoskeleton underlying the membrane^18^. When indenting a bleb (Fig. 4d), the FluidFM probe comes into contact with the membrane after 1.8 s (Fig. 4f). Simultaneously, the Flipper-TR lifetime at the membrane shows a significant increase. After 2.4 s the FluidFM probe enters the plane of view and the intensity at the indentation site rises, while the intensity at the membrane drops, probably due to a positional shift of the whole bleb resulting from its weak cell attachment. Interestingly, in the cytoskeleton-free case of the bleb, an increase in tension occurs instantly at the onset of the mechanical stimulus on the cell membrane at a distance of 5 µm from the stimulation site. Experiments repeated on five separate blebs with an indentation of 50 nN show comparable results (Supplementary Information 8b). This proves that long-range membrane tension propagation is possible in the absence of the cytoskeleton (Fig. 4g,h) and is another indication for the mechanism of tension confinement by cytoskeletal anchors in the membrane.

### Altering cell mechanics affects the *p*_*C,50*_(*F*) curve

To start understanding the influence of the cytoskeleton on membrane tension dynamics and mechanosensation, we chemically modified two cell components. On the one hand, we targeted the actin cortex using cytochalasin D (CytoD), an inhibitor of actin polymerization. On the other hand, the composition of the membrane was altered by adding margaric acid (MargAc), a saturated fatty acid that integrates into the cell membrane.

**Fig. 5:**
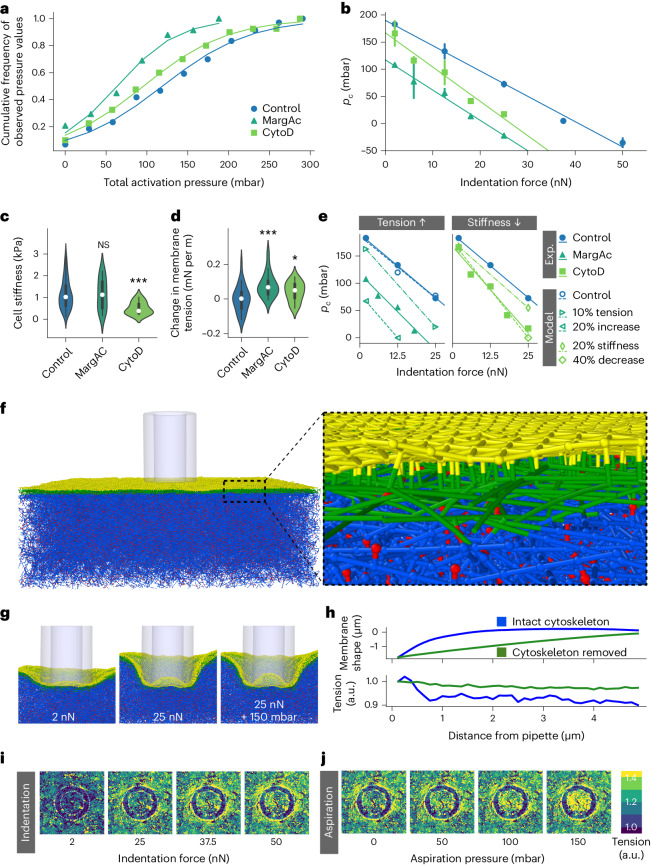
Molecular dynamics model to uncover the role of cytoskeletal components in mechanosensation. **a**, Cumulative histogram of the total activation pressure for different chemical treatments, calculated as the sum of the critical aspiration pressure and indentation force for each individually measured cell, measured in 3 independent biological repeats with a total of 220 cells for Ctrl, 106 cells for MargAc, and 73 cells for CytoD. **b**, Critical aspiration pressure *p*_*C,50*_ as a function of the indentation force *F* for chemical treatment by MargAc and CytoD. Linear fits with *R*^*2*^_Control_ = 0.997, *R*^*2*^_MargAc_ = 0.994 and *R*^*2*^_CytoD_ = 0.980, measured in 3 independent repeats with a total of 25, 24, 27, 10 and 10 cells for MargAc, and 19, 11, 13, 11 and 19 cells for CytoD treatments for the force steps of 2, 6, 12.5, 18 and 25 nN, respectively. The error bars correspond to the standard deviation of the sigmoid fit plus a measure for the goodness of fit of the sigmoidal model, as defined in the Methods section. **c**,**d**, Distribution of cell stiffness (**c**) and cell membrane tension (**d**) for control conditions and upon chemical treatment with MargAc and CytoD, for 142 and 72 cells, respectively. The violin plots show the mean as a white dot, while the black box corresponds to the 25% and 75% quantiles, and the whiskers show the minimum and maximum values. Statistical significance was determined using a Welch’s *t*-test (0.9968 and 0.000009 for cell stiffness measurements, and 0.00081 and 0.02718 for membrane tension measurements). **e**, *p*_*C,50*_(*F*) graph of experimental and modeled data. Model parameters were calibrated using the control case (blue circles), while changes in the modeled data were observed upon increase of the initial membrane tension (left, teal triangles) and decrease of the cytoskeletal stiffness (right, green squares). **f**, Setup of the molecular dynamics model consisting of flexible cytoskeletal filaments (blue), filament interconnections (red), a denser filament cortex below the membrane (green), a flexible membrane with connections to the cytoskeleton (yellow), and the pipette (gray). **g**, Shape of cytoskeleton and membrane at 2 nN and 25 nN indentation depths of the pipette and with an additional 150 mbar aspiration pressure applied. **h**, Shape and normalized tension profile of the membrane at 25 nN indentation with and without underlying cytoskeleton. **i**,**j**, Spatial distribution of local membrane tension changes at the pipette for increasing indentation (**i**) and for 25 nN indentation and increasing aspiration pressures (**j**). Source data

We then assessed the mechanosensitivity of such chemically altered cells via combined indentation and aspiration using the same protocol as in Fig. 3a–c. To compare the overall mechanosensitive response, the total activation pressure was calculated for each cell by summing the combinations measured individually (Supplementary Information 9) for the critical aspiration pressure and indentation force. As seen in the cumulative histogram in Fig. 5a, the total activation pressures needed to elicit a whole-cell calcium response in 50% of the cells, is lowered significantly in both cases, by 25% from 121 ± 5 mbar for Ctrl to 91 ± 5 mbar for CytoD, and by 37% to 76 ± 4 mbar for MargAc-treated cells. In Fig. 5b, this is reflected by a significant downward shift of the *p*_*C,50*_(*F*) curves, by 12% from a *y* intercept of 190 ± 6 mbar for Ctrl to 167 ± 11 mbar for CytoD, and by 38% to 117 ± 5 mbar for MargAc. While the slope of −5.6 ± 0.4 mbar nN^−1^ for the MargAc case lies within 1.5-fold of the confidence interval of the slope of −4.7 ± 0.2 mbar nN^−1^ for the control case, treatment with CytoD leads to a significantly steeper slope of −6.3 ± 0.7 mbar nN^−1^.

To understand these effects on the *p*_*C,50*_(*F*) curve, the changes in cell stiffness and membrane tension were investigated using AFM-based force spectroscopy and Flipper-TR lifetime imaging, respectively. As shown in Fig. 5c, d, CytoD treatment lowered the cell stiffness significantly and slightly increased the resting membrane tension, while MargAc significantly increased the resting membrane tension without changing the cell stiffness, as previously reported^44^.

Given that the mechanosensitive ion channel Piezo1 is triggered by tension, an increased resting membrane tension is expected to increase the number of channels close to the activation threshold. This would cause a decrease in the additional mechanical stimulus needed to elicit a mechanosensitive response. That vertical shift of the *p*_*C,50*_(*F*) curve is therefore associated with the resting membrane tension, while changes in the slope of the *p*_*C,50*_(*F*) curve indicate changes in the cytoskeleton underlying the membrane. This means, for the specific case of the CytoD treatment (Fig. 5b), that cells with inhibited actin polymerization react in almost the same way as control cells at low indentations, but more promptly than control cells at higher indentations. This shows that actin does not play a pivotal role in the response to stimulation by mainly aspiration, but starts playing a mechanoprotective role by hindering the mechanosensitive response at higher indentations. This observation is in agreement with previous reports using whole-cell patch-clamp that CytoD treatment resulted in reduced Piezo ion current amplitudes upon indentation, but not aspiration^18,45,46^. Recent reports of long-range tension propagation upon direct engagement of the actin cortex point towards a stimulus-dependent dual role of the cytoskeleton^12^. Given that FluidFM enables the spatiotemporal combination of indentation and aspiration, it is therefore a powerful method to decouple the influences of the cytoskeleton and the membrane on the process of cellular mechanosensation.

### Molecular dynamics modeling enables interaction studies

To gain an in-depth understanding of how the mechanical components and their properties influence cellular mechanosensitivity, one method is to change the individual cellular components chemically, as described above. However, in many experimental cases, a cross-talk can be observed between different cellular components or biological functions, and effects cannot necessarily be individually and gradually tuned. Therefore, we aimed to create a fully controllable model system to simulate the effects of changes of mechanical cellular components. For this, we set up a coarse-grain particle-based computational model that resembles the modern view of the cytoskeletal and cell membrane organization^47,48^ (see Fig. 5f and Supplementary Information 10 for details on set up and calibration). On the one hand, the actin cortex was mimicked by including a 200-nm-thick layer with higher fiber density just below the membrane. On the other hand, anchored transmembrane fences were included as linkers between the cytoskeleton and the membrane.

The system was disturbed by a hollow pipette of 2 µm in diameter applying increasing indentation and aspiration stimuli, which resulted in a protrusion of the cell membrane formed inside the pipette (Fig. 5g), matching previous experimental observations^5^. When examining the spatial distribution of local membrane tension around the pipette, increasing tension outside and inside the pipette with increasing indentation could be seen (Fig. 5i). Similarly, for increasing aspiration pressures, increasing tension is ascertained inside the pipette, but not outside the pipette (Fig. 5j). These results exhibit the same trend as observed in the experiments in Fig. 2d,e. The effects further from the pipette are shown as a tension profile along a membrane indented with 25 nN (Fig. 5h). For the cytoskeleton-free case, the tension rises throughout the whole membrane. However, with an intact cytoskeleton the tension increase stays confined to a 0.7 µm distance from the indentation site, which is in the range of the 1.7 ± 0.2 μm measured previously^8^. The model thereby confirms the experimental observation from the tension kymographs (Fig. 4), and the anchored transmembrane fence model, suggesting that linkers between the membrane and the cytoskeleton hinder the propagation of membrane tension.

Overall, this model recapitulates all experimental observations and highlights how membrane tension is influenced by the cytoskeleton and the presence of the pipette. Given that the model was constructed by relying solely on the basic understanding of cell structural mechanics, it separately supports our conclusions of the role of individual cell components in the observed processes.

### *p*_*C,50*_(*F*) curve is dependent on cell mechanical properties

The validated model was subsequently used to assess the influence of changes in mechanical cellular components on cellular mechanosensitivity and thereby the trends in the *p*_*C,50*_(*F*) curve. First, the resting membrane tension in the model was increased (Fig. 5e, left). This alteration resulted mainly in a downward vertical shift of the *p*_*C,50*_(*F*) curve, with the vertical shift increasing in the case of a higher initial membrane tension. These results match well with the experimental observations after MargAc treatment, which resulted in both a membrane tension increase and a downward vertical shift of the *p*_*C,50*_(*F*) curve. Second, the cell stiffness in the model was decreased (Fig. 5e, right). For a 40% change in cytoskeleton network parameters, a major steepening in the slope of the *p*_*C,50*_(*F*) curve was observed. These observations are in good agreement with the experimental results obtained after CytoD treatment, showing, on the one hand, a significant decrease in cell stiffness and a small increase in resting membrane tension, while on the other hand, a steeper slope and a slight downward vertical shift of the *p*_*C,50*_(*F*) curve. In summary, both experiments and simulation point towards a mechanism whereby the vertical shift of the *p*_*C,50*_(*F*) reflects the resting membrane tension, while the slope of the *p*_*C,50*_(*F*) curve correlates with the cytoskeletal stiffness.

A higher resting membrane tension leads to more channels close to the activation threshold. Therefore, the vertical shift of the *p*_*C,50*_(*F*) curve is a measure that can be used to compare the mechanosensitive reactivity state of different cell populations to the same controlled stimulus. While this first aspect could be determined from a one-point measurement at a fixed stimulus, measuring the whole *p*_*C,50*_(*F*) curve and deriving its slope opens up a new dimension. Indeed, it offers the possibility to additionally account for the mechanical state of the cell and for the response dependence on the stimulus type, as detailed above for the CytoD case in Fig. 5b. Extracting these features of the mechanosensitive response for different cell populations enables investigation of the role of different membrane and cytoskeletal components, and differences in tension regulation and mechanosensing mechanisms between cell types.

## Discussion

To date, the influence of membrane tension on cell behavior remains incompletely understood. Here, we demonstrated the potential of combining FluidFM stimulation with fluorescent calcium and tension imaging to study the effect of tension dynamics on cellular mechanosensitivity. The experimental system enables the tension to be controlled during pipette–cell contact, the cell-wide mechanosensitive calcium response to be investigated, and the spatial and temporal distribution of membrane tension to be quantified without disruption. In contrast to the patch-clamp, FluidFM measurements can be performed at physiological temperatures, influencing both the mechanical and biochemical components of the cell. The ability to control aspiration pressure and indentation force independently enables the investigation of the cross-talk between cytoskeleton and membrane mechanics^45^, whereby the experimental results can be interpreted on the basis of a detailed molecular dynamics model. Moreover, the sensitive force-control of FluidFM enables the application of both small stimuli down to 1 nN and large ones up to 2 µN, potentially addressing different regimes of the cellular tension machinery^12^.

In our studies we observed a localized increase in tension upon stimulation of the cell membrane, potentially activating a limited number of mechanosensitive ion channels that allow for calcium influx from the stimulation site. A growing field of research is investigating the spatiotemporal dynamics of cell membrane tension and its role in regulating processes including mechanosensation, cell shape and migration, membrane fusion and fission.

The proposed approach offers a direct method to assess the impact of specific cellular treatments on mechanosensation, with unprecedented control of the localization and amplitude of the applied stimulus, either aspiration pressure or indentation force. However, when it is relevant to transduce the information into absolute tension changes, the method relies on the capability of the fluorescent membrane tension probe Flipper-TR. This requires re-calibration of the transduction coefficient for every cell type, which is currently based on the use of an additional technique, optical tweezers. To widen the adoption of the approach across the scientific community, it would be ideal to design a calibration method for Flipper-TR based on FluidFM, that could be adopted in situ.

Moreover, the process of mechanosensing is monitored through the fluorescence of a calcium reporter. The method could be extended to other ions using different reporters, but a direct measurement of the ion current would be more general, as is the case in electrophysiology. While this was shown to be possible using FluidFM technology, there is still a limit in the efficiency of the seal achieved with this technique, which hinders the sensitivity of FluidFM-based electrophysiological measurements^49^.

Finally, while the molecular dynamics model cannot ultimately validate biological processes, in combination with experiments it can give hints on potential mechanisms, for example by carefully changing different structural components such as membrane–cytoskeleton linkers, actin cortex thickness and density, or underlying microtubule density and connectivity. Therefore, the combined application of the experimental and modeling methods presented here will be crucial to gain a deeper understanding of the cell machinery regulating membrane tension and mechanosensitivity.

All in all, the presented method enables a deeper study of mechanosensitive ion channels, leading to insights into the interplay with the local microenvironment^50^ and the impact of membrane–cytoskeleton coupling on the tension propagation and mechanosensitive ion channel activation across the plasma membrane^51^. This tool promises to extend the existing methodological toolbox, and help to address major open questions related to cellular mechanosensation and the mechanobiology of the actin cortex^52^.

## Methods

### Solutions and chemicals

The following solutions and chemicals were used: MEM (Modified Eagles Medium +GlutaMAX, Thermo Fisher Scientific), FBS (Thermo Fisher Scientific), penicillin–streptomycin (Thermo Fisher Scientific), trypsin-EDTA (Thermo Fisher Scientific), PBS (Thermo Fisher Scientific), PDL (poly-d-lysine, Sigma-Aldrich), physiological solution (140 mM NaCl, 5.4 mM KCl, 10 mM HEPES, 10 mM glucose, 1 mM MgCl_2_, 1.8 mM CaCl_2_, pH 7.4 with NAOH, all from Sigma-Aldrich), SR101 (Sulforhodamine 101, Sigma-Aldrich), AMCA (7-amino-4-methylcoumarin, Sigma-Aldrich), PAcrAm-g-(PMOXA, NH_2_, Si) (poly(acrylamide)-*g*-(poly(2-methyl-2-oxazoline),1,6-hexanediamine,3-aminopropyldimethylsilanol), SuSoS), EGTA (ethylene glycol-bis(2-aminoethylether)-N,N,N′,N′-tetraacetic acid, Sigma-Aldrich), Cal520, AM (Abcam), Yoda1 (Sigma-Aldrich), GsMTx4 (Abcam), cytochalasin D (Abcam), margaric acid (heptadecanoic acid, Sigma-Aldrich), sorbitol (Sigma-Aldrich) and Flipper-TR (Spirochrome).

### Cell culture

The cells used were human foreskin fibroblasts (HFF-1, American Type Culture Collection (ATCC)). The cells were cultured under standard adherent cell culture conditions at 37 °C and 5% CO_2_ in MEM culture medium supplemented with 10% FBS and 1% penicillin–streptomycin. They were split using 0.05% trypsin-EDTA and seeded at 5,000 cells cm^−2^ in glass bottom dishes (Willco) 1–2 days before experiments.

For calcium imaging, cells were incubated with 5 µM Cal520 in medium at 37 °C for 60–120 min, subsequently washed, and transferred to physiological solution before experiments, unless otherwise stated. Cell activity and cytoskeletal components were chemically altered by adding 10 µM Yoda for 5 min, 8 µM GsMTx4 for 5 min, 10 µM cytochalasin D for 30 min, or 100 µM margaric acid for 20 h to the bath solution before and during experiments. A calcium-free extracellular environment was created using PBS + 3 mM EGTA as the bath solution. Blebs were formed by adding 4 M sorbitol for 10 min prior to but not during the experiment, as adapted from ref. ^53^.

### Cylindrical FluidFM probe preparation

The FluidFM probes used were custom fabrications at the wafer scale (SmartTip) with a pipette-like tip geometry with a tip length of 10 µm, aperture diameter of 2 µm and cylinder wall thickness of 200 nm. Details of the exact manufacturing process of the cylindrical FluidFM probes used here are described in ref. ^27^, with the only modification being that here no postprocessing by focused ion beam was performed.

For filling and coating, the protocol was adapted from ref. ^29^. In brief, a cylindrical FluidFM probe made of Si_3_N_4_ was plasma treated for 30 s (PDC-002, Harrick Plasma) and the microfluidic channel was filled with physiological solution supplemented with fluorescent dye (during calcium imaging: 0.1 mg ml^−1^ SR101 (red fluorescence); during FLIM imaging: 0.1 mg ml^−1^ AMCA (blue fluorescence)). The probe was then immersed for at least 1 h in 0.05 mg ml^−1^ PAcrAm-g-PMOXA (ref. ^28^) in 1 mM HEPES1 solution, and negative pressure was applied directly after immersion for 20 s to achieve an external and internal non-fouling coating of the probe. The spring constant (nominal value, 1 N m^−1^) and deflection sensitivity were calibrated using optical beam deflection and the Sader method^54–56,57^.

### Combined calcium imaging and mechanical stimulation

Calcium imaging was performed on an AxioObserver.Z1 inverted microscope with a ×40 objective (Carl Zeiss). The dye was excited by an attenuated 470 nm wavelength Colibri LED at low light intensities to avoid an otherwise observed calcium concentration increase solely by light exposure. Images were acquired at a frame rate of 50 ms with an electron-multiplying CCD camera (C9100, Hamamatsu Photonics) that was water cooled externally by a recirculating cooler (F250, Julabo) to avoid vibrations from the camera internal fan cooling.

The mechanical stimulation was performed with a FluidFM system consisting of an AFM system (FlexAFM-near-infrared scan head with a C3000 controller driven by the EasyScan2 software, Nanosurf), a pressure controller with a 1,000 mbar (≙750 mmHg) range (Cytosurge), and a customized motorized *x*–*y* microscopy stage with AFM positioning (Nanosurf). The whole system was placed in an incubator box heated to 37 °C. CO_2_control was not necessary because the cells were measured in a CO_2_-independent buffer for a maximum of 30 min. Timelines of the piezo movement, cantilever deflection and fluidic pressure were recorded using a data acquisition box and custom LabView script (National Instruments), and time correlated to the recorded images by a trigger output from the microscope. For high-resolution imaging, the AFM system and the customized microscopy stage with a stage adapter were placed on a FluoView 3000 inverted confocal laser scanning microscope (Olympus).

The start and propagation speed of calcium concentration increase was determined using a custom Python script (Python v3.6). The stimulation events were segmented by hand, de-noised using Noise2Void (v0.2.1)^58^, and the baseline brightness of each pixel (5th percentile value) was removed, low-pass Gaussian filtered and normalized. From the filtered images, a rise in calcium concentration was detected by threshold and the propagation speed was determined from the traveled distance of the wave front over 50 frames.

### Single-cell mechanical stimulation

The FluidFM probe was positioned 10 µm above the glass surface and a cell was positioned underneath. Contact between cell and probe was established in the force spectroscopy mode by approaching the cell at 1 µm s^−1^ until the desired contact force of between 2 nN and 50 nN was reached, and the contact force was subsequently held constant by the AFM PID (proportional–integral–derivative) controller in standard ‘contact mode’. After establishment of the contact, aspiration pressure pulses from 0 to 400 mbar (≙400 mmHg) in steps of −25 mbar (≙ −18.75 mmHg) were applied through the microchannel of the cantilever with a duration of 5 s and pauses at 0 mbar of 1 s. After the increase of calcium concentration in the cell at the critical aspiration pressure, the pressure was set to zero, the probe was removed from the cell and the microchannel was cleaned by a pulse of 1,000 mbar (≙750 mmHg) overpressure. On every cell, only one measurement was conducted to avoid effects from previous activation.

For each cell, the measured critical aspiration pressure as a function of the applied indentation force was processed as follows: a cumulative histogram of the critical aspiration pressures was collected for each indentation force and chemical treatment (Supplementary Information 9). To these data a sigmoidal curve with formula $1/(1+{e}^{-(x-a)/b})$ was fitted to extract *p*_*C,50*_ as the pressure of half-maximum response. The obtained *p*_*C,50*_ values were then plotted versus the indentation curve to obtain the *p*_*C,50*_(*F*) curve from which the vertical shift and slope can be inferred for each cell population by a least squares linear fit. The error of the *p*_*C,50*_ value for each indentation force is determined as ${{\mathrm{error}}}=\sigma +\frac{{p}_{C,50}}{{{\mathrm{GoM}}}}$, with σ as the standard deviation of the sigmoidal fit and GoM as the goodness of fit of the sigmoidal model compared to a linear fit, defined as ${{\mathrm{GoM}}}=\frac{{\chi }_{{{\mathrm{lin}}}}^{2}}{{\chi }_{{{\mathrm{sig}}}}^{2}}+1$, with ${\chi }_{{{\mathrm{lin}}}}^{2}=\sum _{{p}_{C}}({{{\mathrm{Cumulative}}\; {\mathrm{frequency}}}-{{\mathrm{Expected}}\; {\mathrm{value}}\; {\mathrm{from}}\; {\mathrm{linear}}\; {\mathrm{fit}}}})^{2}$ and ${\chi }_{{{\mathrm{sig}}}}^{2}=\sum _{{p}_{C}}({{{\mathrm{Cumulative}}\; {\mathrm{frequency}}}-{{\mathrm{Expected}}\; {\mathrm{value}}\; {\mathrm{from}}\; {\mathrm{sigmoidal}}\; {\mathrm{fit}}}})^{2}.$ Then, to obtain the total activation pressure, the values for each cell are treated individually. First, the indentation force is converted into pressure using the slope obtained from the *p*_*C,50*_(*F*) curve. The total pressure for each cell is then calculated by summing the critical aspiration pressure and the converted indentation force. Upon plotting these total pressures in a cumulative histogram, a sigmoidal curve is then fitted to these data to extract the total activation pressure of the cell population as the pressure of half-maximum response.

Cell stiffness values were extracted from the FluidFM-based force spectroscopy performed during each cell approach in the protocol described above. The cell stiffness was extracted from the recorded force–indentation curves with a custom Python user interface as described previously^59^. In brief, the contact point of the force–distance curve was determined by thresholding, and the Hertz model was fitted to the force–indentation curve to obtain the cell stiffness.

### Combined FLIM imaging and mechanical stimulation

To enable the combination of FluidFM stimulation with FLIM imaging, several adjustments were made to the microscope and the AFM scan head, as detailed in Supplementary Information 1. Once established, FLIM imaging was performed in the presence of the FluidFM probe on cells incubated with 1 µM Flipper-TR for 10 min. FLIM imaging was carried out on a Leica SP8 inverted confocal laser microscope run by the LAS X software (Leica) with a ×63 objective (HC PL APO CS2 ×63/1.40 OIL, Leica), and excitation with a white light laser at 20% laser power and 488 nm. Whole-cell images were taken with a zoom factor of 1, pixel dimensions of 361 nm, a scan speed of 400 Hz, and 23× frame averaging. For images of the tube tip, a zoom factor of 7.24, pixel dimensions of 100 nm, a scan speed of 400 Hz, and 30× frame averaging were used. Single photons and their lifetimes between 550 nm and 800 nm were collected with a HyD detector in counting mode. For imaging at the indentation site, the focal plane was adjusted to the tip of the FluidFM probe after contact with 2 nN indentation force and an aperture of 3 Airy units was chosen. Due to the large optical aperture, the tube tip remained in focus during further indentation. For zoomed images without indentation, the focus plane was set to the highest point of the upper cell membrane.

For statistically relevant lifetime determination, a minimum of 1,000 photons per pixel were collected and a 2-exponential reconvolution fit was performed to the time-correlated single-photon counting histogram (Fig. 2b, top)^40,41^. Hereby, the two components of the Flipper-TR lifetime were determined using the LAS X software (Leica), and the smaller component was fixed for each image and neglected for all further analyses. The larger component with direct relation to the membrane tension^26^ was determined for each pixel, yielding the spatial distribution of membrane tension during mechanical stimulation. By thresholding the fluorescence intensity of each pixel (Fig. 2c, middle), membrane areas were separated from the background and the average fluorescent lifetime for a selected region of interest was determined. To obtain tension kymographs, line imaging over time was performed and at least 500 photons per pixel were collected in 16 line repetitions, resulting in a time resolution of 20–80 ms per line. During imaging, cells or blebs were indented with 7.5 nN or 50 nN. The obtained fluorescence lifetimes are converted to membrane tension using the conversion factor of 1.325 (mN m^−1^) ns^−1^ as calibrated by tether pulling (see below).

### Calibration of Flipper-TR lifetime versus membrane tension in HFF-1 cells

HFF-1 cells were cultured and detached as described above, subsequently seeded in a glass bottom plate in physiological solution and 1 μM Flipper-TR, and left to adhere for 30–60 min at 37 °C. The osmolarity pressure was then changed by adding adjusted mixtures of physiological solution and Milli-Q water to the well. The osmolarity of the solutions was measured separately with an osmometer. Flipper-TR lifetimes of a total of 21–23 cells per condition in three separate dishes were measured at 37 °C as described above, using the same imaging and analysis parameters. Membrane tension was measured for 7–10 cells per osmotic condition by pulling membrane tethers attached to Concanavalin A-conjugated beads using a custom optical setup combining optical tweezers and imaging^60^.The membrane tension was obtained by tether force at the plateau as described in ref. ^61^, and more details are given in Supplementary Information 2.

### Coarse-grained molecular dynamics model

The coarse-grain particle-based model of a portion of the cell was based on the previously developed eukaryotic cell model^48^. The simulations were performed using LAMMPS^62^ at Swiss National Supercomputer Center (CSCS). The model has three main components: cell membrane, cortex and cytoskeleton. The membrane consists of particles with flexible connections forming a surface triangulation. The cytoskeleton consists of a random network of filaments that are assembled from particles connected with links. The cortex is composed of the same filaments but exhibits higher density and a preferred orientation of the filaments along the membrane. All components are placed in a fluid environment and connected to each other by cross-links. The micropipette is modeled using solid wall boundary conditions, and adhesion of the membrane to the pipette is imposed. More detailed information on the setup and calibration of the model is given in Supplementary Information 10.

### Statistics and Reproducibility

No statistical method was used to predetermine sample size, but a minimum of three independent replicates were performed for all experiments. Unless otherwise stated, individual values were aggregated by calculating the mean and standard deviation or standard error as reported in the figure legends. Statistical significance was determined using a two-sided *t*-test. For the mechanical stimulation of cells, data were excluded when the blockage of the FluidFM probe or a rupture of the cell membrane occurred, as determined by release or cell entry of SR101, respectively. The experiments were not randomized and the investigators were not blinded to allocation during experiments or outcome assessment.

### Reporting summary

Further information on research design is available in the Nature Portfolio Reporting Summary linked to this article.

## Online content

Any methods, additional references, Nature Portfolio reporting summaries, source data, extended data, supplementary information, acknowledgements, peer review information; details of author contributions and competing interests; and statements of data and code availability are available at 10.1038/s41592-024-02277-8.

### Supplementary information

Supplementary Information Supplementary Information 1–10 containing Supplementary Figs. 1, 2, 3, 4, 5, 7, 8, 9 and Supplementary Discussion 2, 3, 4, 6, 10.

Reporting Summary

Supplementary Video 1 Calcium response of intact cell.

Supplementary Video 2 Calcium response of ruptured cell.

Supplementary Video 3 Calcium response upon high indentation.

### Source data

Source Data Fig. 1 Statistical source data.

Source Data Fig. 2 Statistical source data.

Source Data Fig. 3 Statistical source data.

Source Data Fig. 4 Statistical source data.

Source Data Fig. 5 Statistical source data.

## Data Availability

Full data sets are available in the data repository published here: 10.3929/ethz-b-000659903 (ref. ^42^). The data repository includes complete image data sets for the step-wise and real-time measurement of Flipper-TR. For the calcium imaging data, representative image files are included, due to the large size of the full data set. The full data set for calcium imaging is available from the corresponding authors upon request and a file including all extracted datapoints are included in the repository. Source data are provided with this paper.

## Code availability

The computational model that is the basis for the molecular dynamics model was previously peer-reviewed and published in *PLOS Computational Biolog*y^48^, and the implementation is based on the open-source Large-scale Atomic/Molecular Massively Parallel Simulator, LAMMPS, which is available together with information on its installation and usage at www.lammps.org.
