# Supplementary material for: Dissecting cell membrane tension dynamics and its effect on Piezo1-mediated cellular mechanosensitivity using force-controlled nanopipettes
Source: Nat Methods. 2024 May 27;21(6):1063–73. doi: 10.1038/s41592-024-02277-8 (PMC11166569; doi:10.1038/s41592-024-02277-8)
Supplement: Supplementary file 1 — Supplementary Information 1–10 containing Supplementary Figs. 1, 2, 3, 4, 5, 7, 8, 9 and Supplementary Discussion 2, 3, 4, 6, 10. [file 41592_2024_2277_MOESM1_ESM.pdf]

nature methods

Supplementary information https://doi.org/10.1038/s41592-024-02277-8

Dissecting cell membrane tension dynamics and its effect on Piezo1-mediated cellular mechanosensitivity using force-controlled nanopipettes

In the format provided by the authors and unedited

<u>SV_1:</u> Video of FluidFM stimulation and calcium response of the cell

Real-time video showing the positioning of FluidFM cantilever on top of cell, stimulation by first 25 nN indentation, then 200 mbar aspiration, and the subsequent calcium response of the cell, followed by check for membrane rupture in the red channel.

Time (min:sec)	Experimental Step + Observation
00:00	HFF1 cell positioned in center of image, exhibiting low green fluorescence of Cal520 calcium sensitive dye. FluidFM cantilever (dark rectangle in top center) positioned such, that the tip aperture is in the center of the image, and central but next to the nucleus.
00:01	Downward movement of cantilever (not seen, since the movement occurs in vertical direction), indentation of the cell up to 25 nN. No calcium response observed.
00:04	Application of aspiration stimulus of 200 mbar. Start of fluorescence increase at site of stimulation, corresponding to an intracellular increase in calcium concentration.
00:09	Spreading of calcium throughout the whole cell, here defined as a whole-cell mechanosensitive response. FluidFM cantilever staying in contact at 25 nN with aspiration pressure of 200 mbar continuously applied.
00:12	Slow decrease of calcium signal in the cell.
00:18	Switch to red fluorescent channel. Aspiration pressure is switched off. The cantilever releases the contact and moves to about 10 µm above the cell.
00:23	An overpressure of 100 mbar is applied to the channel of the FluidFM probe for cleaning of possible cell debris. Thereby, releasing from the tip aperture the filling solution containing the non-membrane permeable red fluorescent dye sulforhodamine 101 (SR101).
00:25	Spreading of SR101 through the field of view, but no entry into the cell. Therefore, the cell membrane is classified as intact, no rupture occurred, and the observed green fluorescent calcium response is counted as a whole-cell mechanosenstive response.

<u>SV_2:</u> Video of cell membrane rupture of upon stimulation by FluidFM

Real-time video showing the positioning of FluidFM cantilever on top of cell, stimulation by first 25 nN indentation, then 200 mbar aspiration, and the subsequent calcium response of the cell, followed by check for membrane rupture in the red channel.

Time (min:sec)	Experimental Step + Observation
00:00	Sparsely seeded HFF1 cells exhibiting low green fluorescence of Cal520 calcium sensitive dye. FluidFM cantilever (dark rectangle in top center) positioned such, that the tip aperture is in the center of the image, above the centrally located cell.
00:03	Downward movement of cantilever, indentation of the cell up to 25 nN. No calcium response observed.
00:08	Application of strong aspiration stimulus of 800 mbar. Start of fluorescence increase at site of stimulation, corresponding to an intracellular increase in calcium concentration.
00:10	Spreading of strong calcium signal throughout the whole cell. FluidFM cantilever staying in contact at 25 nN with aspiration pressure of 800 mbar continuously applied.
00:14	Strong increase of calcium levels in neighboring cells, characteristic for events of membrane rupture of stimulated cell.
00:15	Aspiration pressure is switched off. The cantilever releases the contact and moves to about 10 µm above the cell. An overpressure of 100 mbar is applied to the channel of the FluidFM probe for cleaning of possible cell debris. Thereby, releasing from the tip aperture the filling solution containing the non-membrane permeable red fluorescent dye sulforhodamine 101 (SR101).
00:17	Switch to red fluorescent channel. The shape of the stimulated cell can be noticed in red in the center of the image, indicating that SR101 entered the cell and a membrane rupture occurred. Such experimental cases were not counted as mechanosensitive responses and excluded from further analysis.

<u>SV_3:</u> Video of direct calcium response of a cell at high indentation stimulus

Real-time video showing the positioning of FluidFM cantilever on top of cell, stimulation by 100 nN indentation, and the subsequent calcium response of the cell, followed by check for membrane rupture in the red channel.

Time (min:sec)	Experimental Step + Observation
00:00	HFF1 cell positioned in center of image, exhibiting low green fluorescence of Cal520 calcium sensitive dye. FluidFM cantilever (dark rectangle in top center) positioned such, that the tip aperture is in the center of the image, and central but next to the nucleus.
00:01	Downward movement of cantilever (not seen, since the movement occurs in vertical direction).
00:03	Contact of FluidFM cantilever of 100 nN. Start of fluorescence increase at site of stimulation, corresponding to an intracellular increase in calcium concentration.
00:04	Spreading of calcium throughout the whole cell. FluidFM cantilever staying in contact at 100 nN.
00:16	Switch to red fluorescent channel. The cantilever releases the contact and moves to about 10 µm above the cell.
00:17	An overpressure of 100 mbar is applied to the channel of the FluidFM probe for cleaning of possible cell debris. Thereby, releasing from the tip aperture the filling solution containing the non-membrane permeable red fluorescent dye sulforhodamine 101 (SR101).
00:20	Spreading of SR101 through the field of view, but no entry into the cell. Therefore, the cell membrane is classified as intact, no rupture occurred, and the observed green fluorescent calcium response is counted as a whole-cell mechanosenstive response.

SI_1: Setup details for combining FluidFM with FLIM imaging.

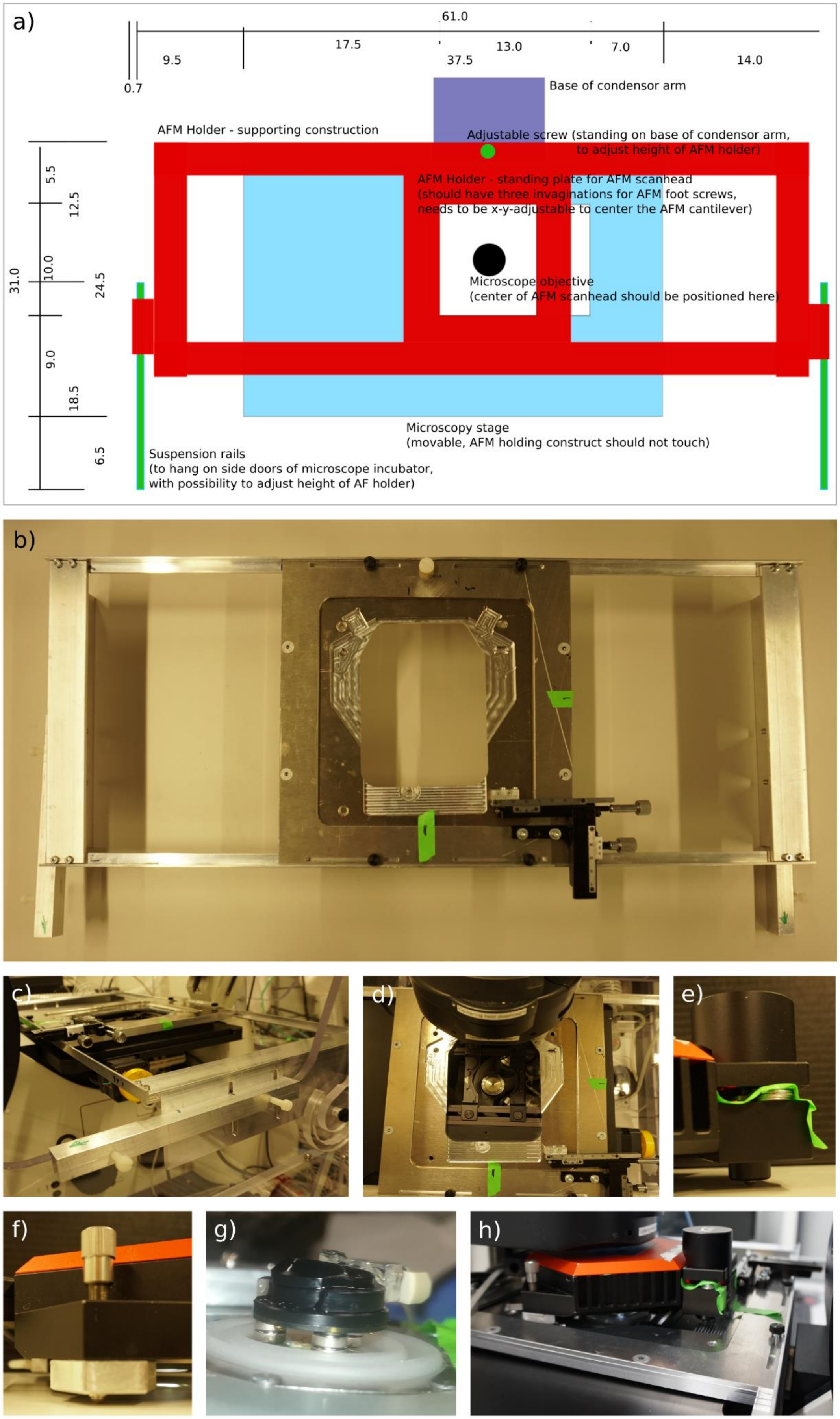

Modifications of microscopy stage and AFM scanhead to enable the combination of FLIM imaging and FluidFM stimulation.
a) Sketch of custom scanhead holder for Leica SP8 inverted microscope and a custom incubator box
b) Custom-made AFM scanhead holder with micro-scale positioning system for scanhead (middle) and attachment rails (sides)
c) Position of attachment rails on doors of microscopy incubator for contactless placement of scanhead holder above microscopy stage
d) Position of scanhead holder above microscopy setup
e) Extension of AFM front screw by increasing the motor position
f) Lowering of AFM back screws by exchanging screw threads
g) Lowering of AFM cantilever by placing magnets between scanhead and cantilever holder
h) AFM scanhead on top of microscopy setup.

<u>SI_1 - Method:</u> Modification of AFM scanhead and holder for combination with FLIM imaging

To enable the combination of FluidFM stimulation with FLIM imaging, several adjustments were made to the microscope and the AFM scanhead, as detailed in the following text and figure. Firstly, on the SP8 FALCON inverted confocal microscope (Leica Microsystems GmbH, Wetzlar, Germany), the condenser lens and the z-galvo stage were removed from the microscope. For positioning of the FluidFM probe independent of sample movements, the AFM scanhead was placed above the standard microscopy stage with a custom-built mount (SI1a-d). This mount was attached to the microscope’s incubator box and adjusted to hover around 0.5 mm above the microscopy stage without contact. The integrated manual x-y-positioning system allowed for sample-independent stable and precise positioning on the FluidFM probe in the field of view. For the FluidFM probe to reach the substrate surface despite of the AFM scanhead’s elevated positioning, several modifications were necessary (SI_1e-g). Firstly, the AFM z-screw holders and z-motor housing were adjusted to allow for extended lowering of the scanhead. Secondly, magnets were placed between the scanhead and cantilever holder to additionally lower the FluidFM probe by around 5 mm, but still allow for AFM laser alignment. Lastly, the sample was placed on top of spacer at the most elevated position which still allowed for microscopy access. The AFM laser alignment was monitored by an ocular camera (DinoEye, AnMo Electronics Co, Taipei, Taiwan).

<u>SI_2</u>: Tension calibration of Flipper-TR lifetimes in HFF1 cells

a)

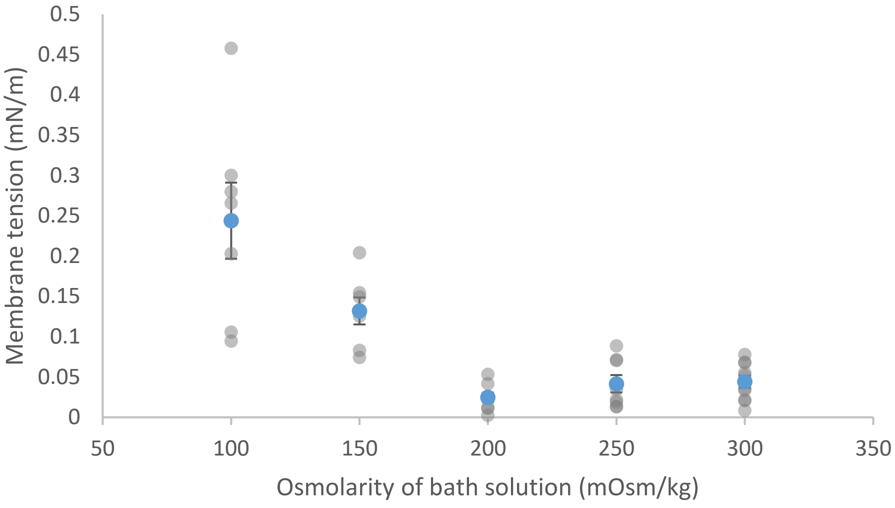

b)

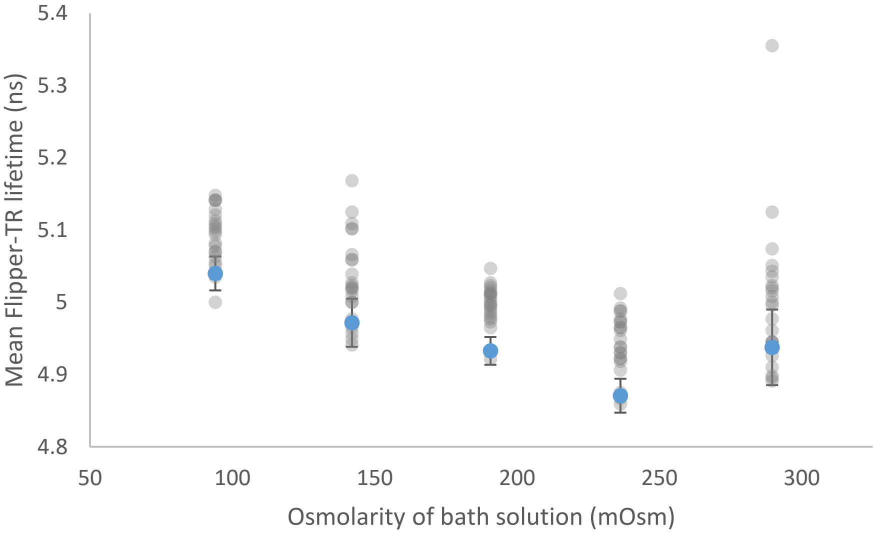

c)

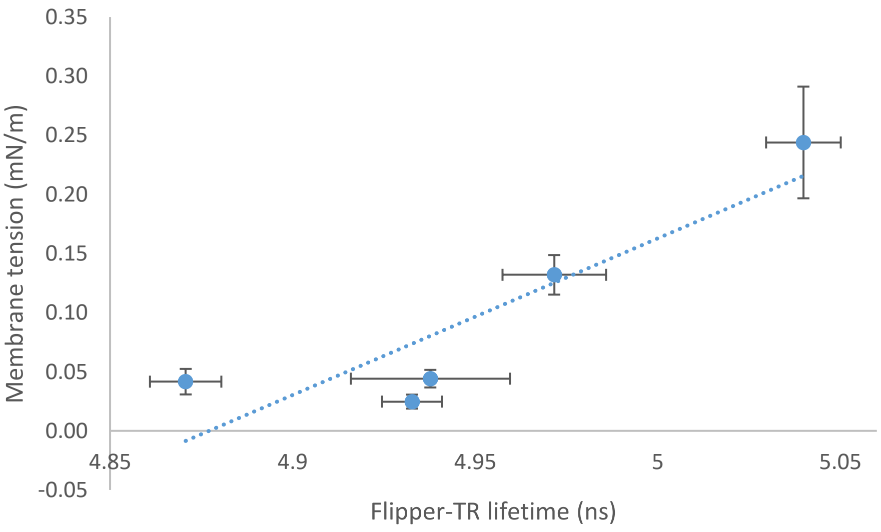

a) Average membrane tension as a function of the osmolarity of the bath solution, as measured by optical trap tether pulling for 7, 7, 8, 8, and 10 cells for the osmotic conditions of 100, 150, 200, 250,and 300 mOsm/kg, respectively. Error bars correspond to one standard error.
b) Average Flipper-TR lifetime as a function of the osmolarity of the bath solution, as measured for 21, 22, 22, 23, and 23 cells for the osmotic conditions of 100, 150, 200, 250,and 300 mOsm/kg, respectively. Error bars correspond to one standard error.
c) Membrane tension as a function of the mean Flipper-TR lifetime, as determined from graphs a) and b). Error bars correspond to one standard error.

SI_2 - Method: Tether pulling experiments and analysis

For tether pulling experiments, HFF1 cells were cultured as described in the main methods subsection "Calibration of Flipper-TR lifetime versus membrane tension in HFF1 cells".
A mixture of HFF1 cells and Concanavalin A conjugated beads [1] was seeded in a glass bottom plate in physiological solution and let to adhere for 30 min at 37°C. The osmolarity pressure was then changed by adding adjusted mixtures of physiological solution and mQ water to the well directly on the microscope prior to starting the measurements. Tether pulling experiments were performed at 37°C on 7 to 10 cells per condition on a custom optical setup combining optical tweezers and imaging [2].
Membrane tethers were pulled from single adherent cells with 2 µm Concanavalin A conjugated 2-µm latex beads as handles. Single beads were trapped in solution and put in contact with the cell membrane. Tether elongation was achieved by moving the microscope stage at 0.5 µm/sec to displace the cell from the trapped bead [3].
For analysis, measurements were selected in which the tether force reached a constant plateau and, after that, the tether broke and the trapped bead recovered to its resting position. In this way, the tether force F_0 was clearly determined from the difference between the force before and after tether rupture. The force was measured along the three spatial directions (F_{x0}, F_{y0}, F_{z0}) using a quadrant photodiode placed in the back focal plane of the condenser. Tether force F_0 was obtained from the bead position and trap stiffness after trap calibration using the power spectrum method [4]. The membrane tension T was then obtained as: $T = \frac{F_0^2}{8\pi^2 B}$
with $F_0 = \sqrt{Fx_0^2 + Fy_0^2 + Fz_0^2}$ and cell membrane bending rigidity B = 0.14 pN·µm as in Colom et al. [1].

SI_2 - Discussion: Measured range of tension values

Previous studies reported values of 1.28 ± 0.23 (mN/m)/ns and 3.85 ± 0.89 (mN/m)/ns for HeLa cells in the low and high tension regime, respectively, while for MDCK cells 0.42 ± 0.03 (mN/m)/ns and 5.1 ± 1.8 (mN/m)/ns were measured for low and high tensions, respectively [1]. The coefficient measured for HFF1 lies in the same range and confirms the strong dependence of this parameter from the cell type and tension regime. This result supports the importance of performing the calibration for each cell type, to properly compare the experimental values, and it highlights a natural variability that can be partly associated to differences in the resting membrane tension between cell types. In fact, previous studies reported tether forces of 7 pN for fibroblasts, 13 pN for HeLa cells, 50 pN for MDCK cells, and 15 pN for HEK293 cells [5][6], where the tether force is proportional to membrane tension. The biological variability observed in tether forces is expected to be reflected in the conversion factor, with lower resting tension being associated to smaller tension changes upon stimulation (and therefore a lower conversion factor). It is important to note, that all findings reported in the presented work are drawn from direct comparisons between equally stained and imaged conditions, and therefore the qualitative behavior does not depend on the conversion factor between Flipper-TR lifetime and membrane tension, which is instead crucial when comparing with different cell types.
The tension changes measured in Fig2f+h are smaller than previously reported tension measurements on other cell types of 1.35 mN/m [1], but correspond to this cell type's physiological range as they are larger than the tension change of 0.2 mN/m observed for a strong osmotic shock of 100mOsm (see SI3). If required, there is no direct limit to applying larger stimuli with the proposed method, as shown by the exemplary measurement up to 300 nN in SI6c and discussed in more detail in SI6d.

<u>SI_3:</u> Laurdan imaging to determine changes in lipid order during mechanical stimulation

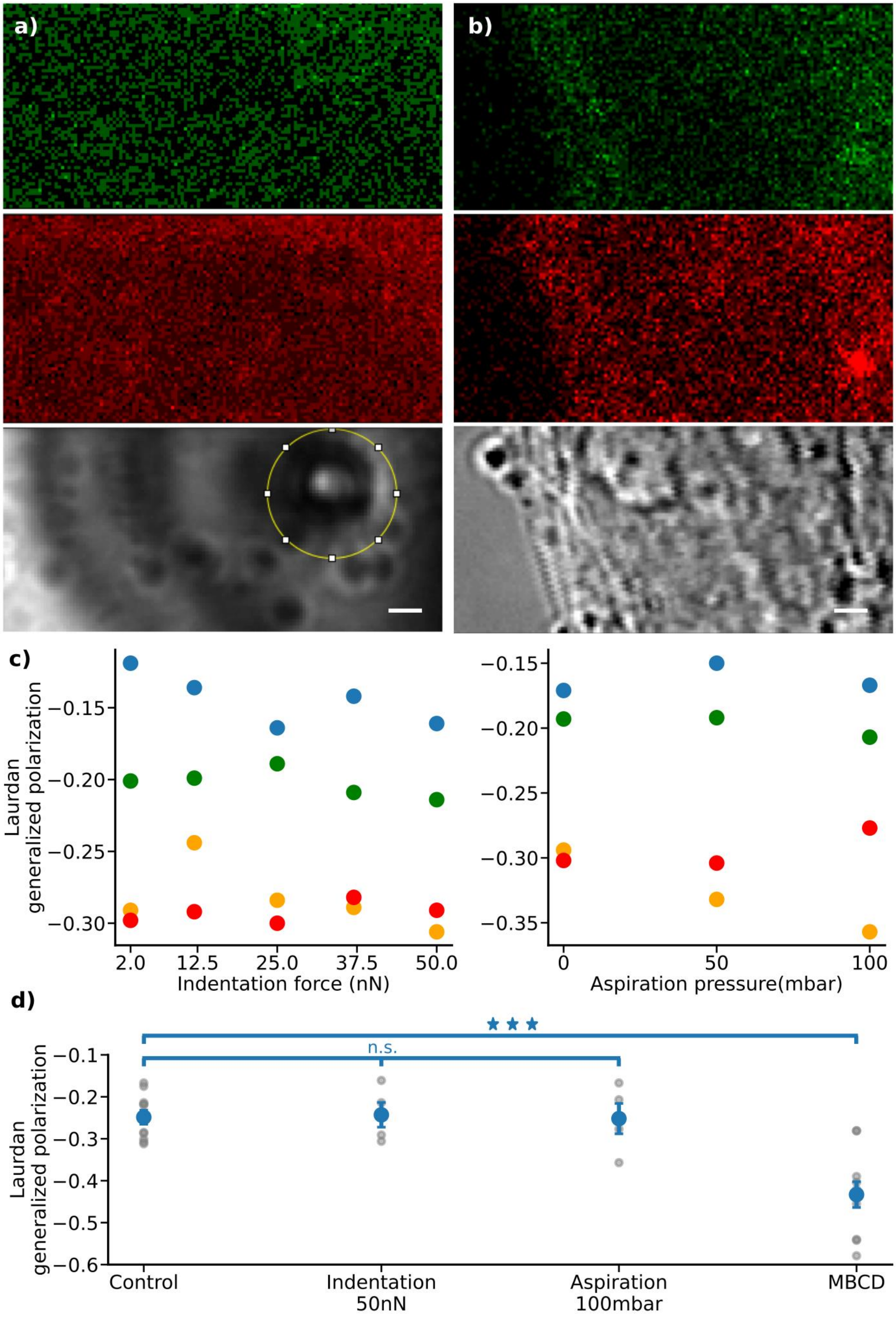

a) Example image of a cell being indented with a FluidFM probe (see brightfield image, bottom) while measuring the Laurdan emission intensity between 410 to 460 nm (see green pseudocoloring, top) and 470 to 520 nm (see red pseudocoloring, center). The yellow ring depicts the area in which the fluorescence intensity was measured in each channel. Scale bar corresponds to 1 µm.
b) Example image of a cell after MBCD treatment, imaged as stated in a). Scale bar corresponds to 1 µm.
c) Change of the generalized polarization of Laurdan fluorescence during increasing indentation (left) and aspiration (right), as measured for 4 cells.
d) Generalized polarization of Laurdan fluorescence of HFF1 cells after different mechanical stimulations or chemical treatment, as measured for 4 cells for indentation and aspiration, and 10 cells for control and MBCD treatments. Blue markers depict the mean, and error bars correspond to one standard error. Significance levels were determined using a 2-sided t-test to $p_{indentation}$=0.87, $p_{aspiration}$=0.92, p_{MBCD}=0.00009.

SI_3 - Method: Laurdan imaging during mechanical stimulation

For imaging of lipid phase transitions in the cell membrane during mechanical stimulation, HFF1 cells were cultured as described in the main methods section and incubated with 5 µM Laurdan (Avanti Polar Lipids, Birmingham, US) for 30 min at 37°C in culture medium, with subsequent washing and change to physiological solution for experiments at 37°C. For chemical alteration, the cells were simultaneously incubated with 10 mM of the cholesterol-depleting agend MBDC (methyl-beta-cyclodextrin, Sigma-Aldrich, St. Louis, USA) for 30 min. For mechanical stimulation, the cells were subjected to increasing indentation, or indentation at 25 nN and increasing aspiration by FluidFM as described in the main methods section. Imaging was performed with the same microscopy setup and general settings as the FLIM imaging described in the main methods section. The focus plane was either set to the top membrane of each cell, or to the tip of the FluidFM probe. To determine the spectral shift of Laurdan, the dye was excited at 405 nm, and its emission intensity was measured separately as I_1 in channel 1 at 410 to 460 nm and as I_2 in channel 2 at 470 to 520 nm. While signal I_1 corresponds to more ordered lipid domains, signal I_2 corresponds to more disordered lipid domains. Potential shifts from one state to the other were quantified by calculating the generalized polarization as $GP = (I_1 - I_2)/(I_1 + I_2)$. For image analysis, in chemical cases the membrane area of the whole cell was taken into account, while for mechanical cases only the membrane area around the FluidFM probe was analyzed.

SI_3 – Discussion: Laurdan imaging during mechanical stimulation

Since Flipper-TR lifetime can not only be affected by membrane tension, but also by lipid order [1], it was investigated how the distribution of lipid phases in the cell membrane is altered during mechanical stimulation. For this, the spectral shift of the lipid phase sensitive fluorescent dye Laurdan [7] was measured at the indentation site and converted into generalized polarization (GP). As seen in SI4, for HFF1 cells neither indentation nor aspiration affect the average GP value, while cholesterol depletion with MBCD decreases it. Therefore, it was concluded that the observed changes in Flipper-TR lifetime are mostly associated to membrane tension increase and not to changes in lipid order.

<u>SI_4:</u> Mechanosensitive response of cells for indentation at higher forces

a)

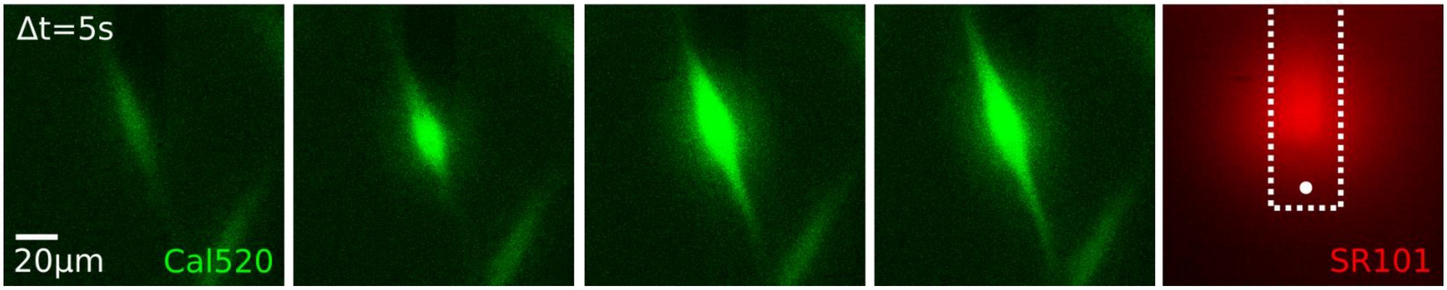

b)

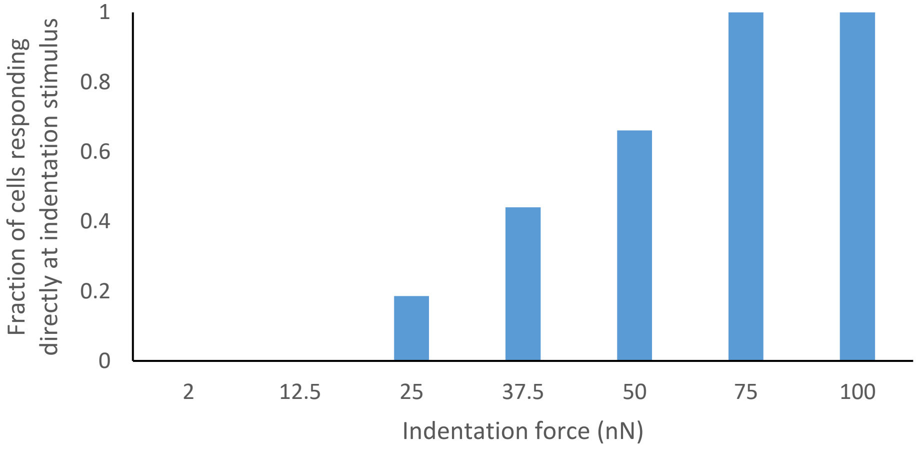

c)

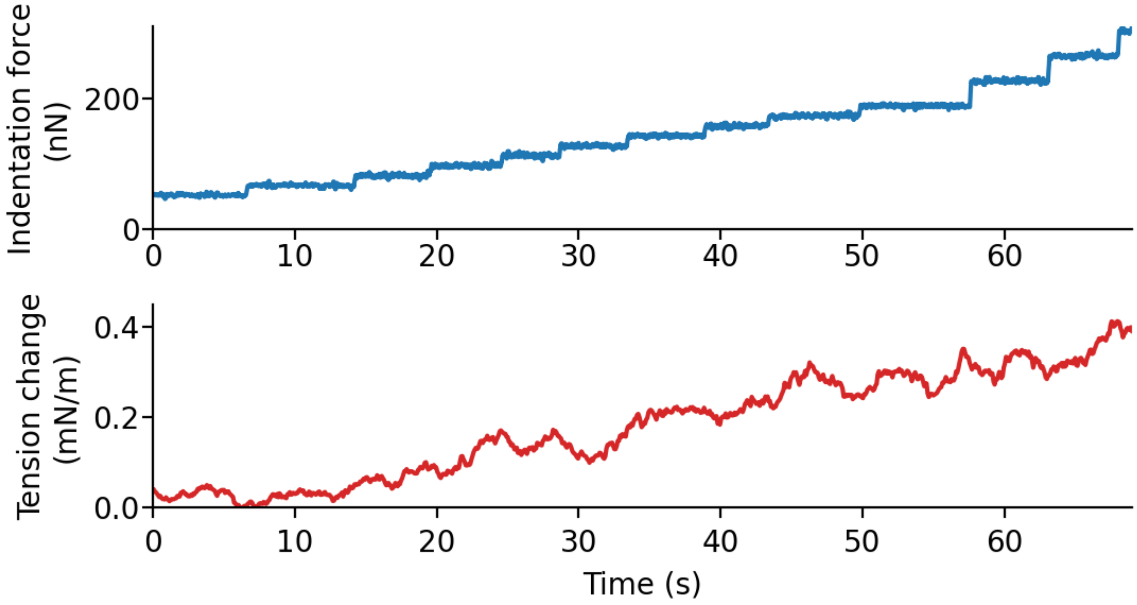

a) Representative time series of a HFF cell stimulated with 100 nN indentation. The green channel shows the cell permeable calcium sensitive dye Cal520-AM. The red channel shows the membrane impermeable dye sulphorhodamine 101 (SR101), that is added to the pipette solution (scale bar: 20 µm).

b) Fraction of cells responding directly to various indentation forces, without subsequent aspiration. The measurement was performed in 3 independent experiments on a total number of 60, 24, 43, 34, 59, 5, and 46 cells for the increasing indentation forces, respectively.

c) Realtime tension measurement on a cell indented with increasing indentation forces up to 300 nN.

<u>SI_4 – Discussion:</u> Applicability of the proposed method to larger mechanical stimuli

FluidFM provides the necessary force control and sensitivity to address the both small and large stimuli, and thereby study different regimes of the cellular tension machinery. The main advantage of the proposed method lies in the control of small stimuli that affect only the cell membrane without impacting other cellular organelles. Nevertheless, as seen in the exemplary measurements in SI6a-c, the proposed method also offers the possibility to apply larger stimuli, with AFM and FluidFM indentation ranges going up to >500 and >2000 nN [8][9], and FluidFM enabling aspiration pressures of up to 800 mbar [10][11] compared to the <150 mbar typically applied in micropipette aspiration assays. Additionally, Flipper-TR has been shown to measure tension changes in cells of up to 1.8 ns and 1.35 mN/m. However, in the assay presented here, we observed that going above 50 nN indentation elicited a direct calcium response to indentation without subsequent aspiration pulses in 100% of the cells (see SI6a+b and SV3), achieving a plateau response and therefore rending this stimulation range unsuitable for the question addressed here.

SI_5: Indentation depth vs. indentation force

a)

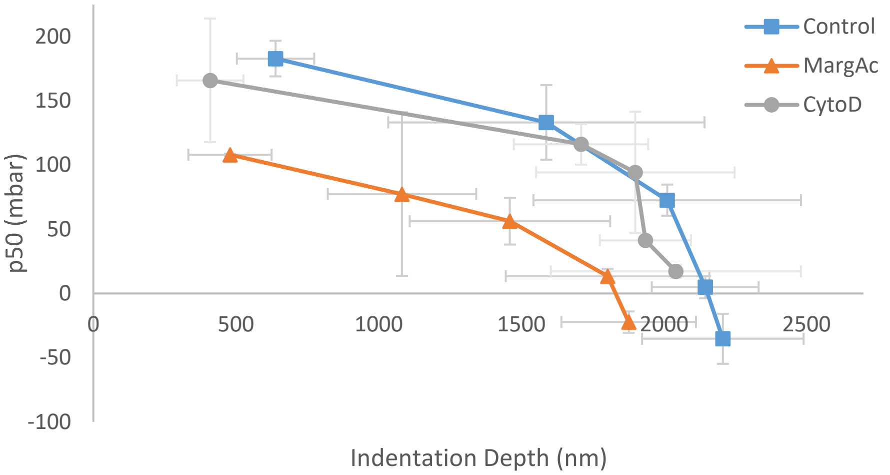

b)

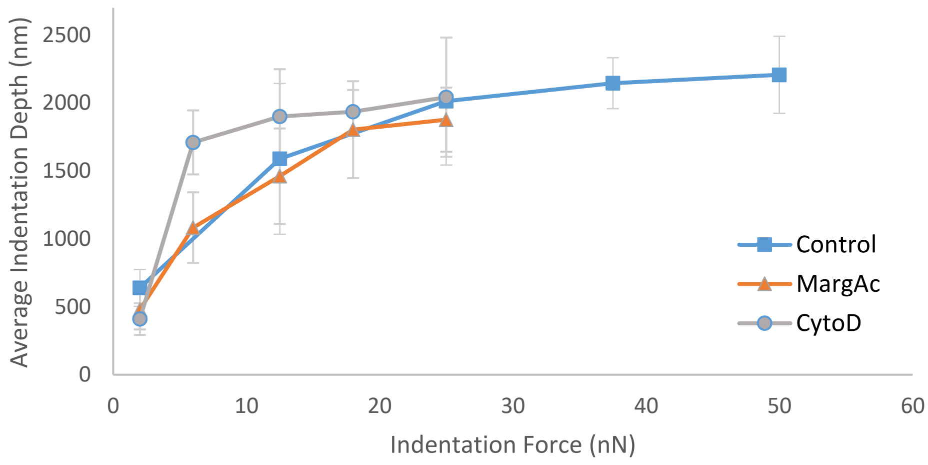

c)

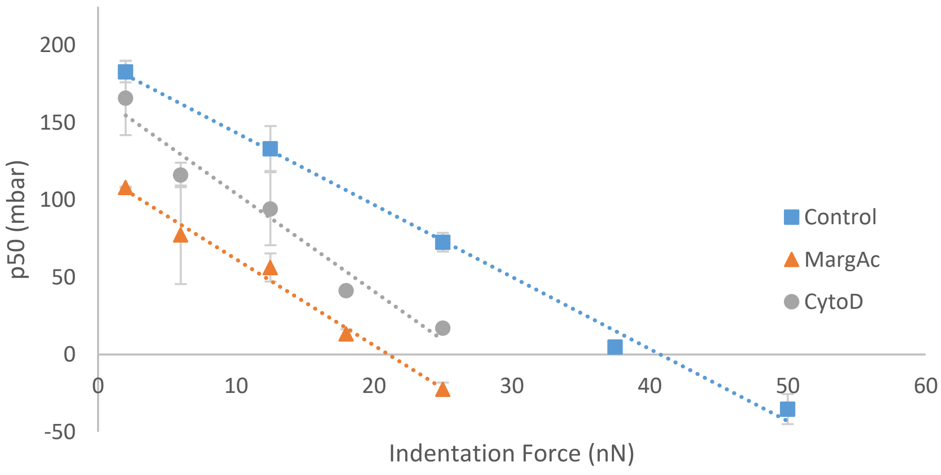

a) Critical aspiration pressure $p_{C,50}$ as a function of indentation depth.

b) Average indentation depth as a function of indentation force.

c) Critical aspiration pressure $p_{C,50}$ as a function of indentation force, for the control case and cell treatment with margaric acid and cytochalasin D.

All data results from the data measured in Fig5b in three independent replicates with a total of 60, 24, 43, 34, and 59 cells for the force steps of 2, 12.5, 25, 37.5 and 50 nN, respectively in the control case, and of 25, 24, 27, 10 and 10 cells for MargAc, and 19, 11, 13, 11 and 19 cells for CytoD treatments for the force steps of 2, 6, 12.5, 18 and 25 nN, respectively. The error bars correspond to the standard deviation of the sigmoid fit plus a measure for the goodness of the sigmoidal model, as defined in the Methods section.

SI_6 Discussion: Comparison of stimuli magnitudes to previous literature

While not directly comparable, the maximal stimuli of 50 nN indentation and 181 mbar (≙ 136 mmHg) aspiration required with this method to elicit a mechanosensitive response by only indentation or aspiration, respectively (see Fig3c), are compatible with previous results. AFM-based measurements on HEK293 cells [8] showed a much higher force threshold ≤200nN, associated with larger contact area of a large solid sphere compared to a hollow FluidFM cantilever, resulting in the lower accessibility of calcium ions to the membrane and ion channels. Micropipette-based experiments conducted on HEK293 cells generally showed pressure values of ≤ 80 mbar (≙ 60 mmHg) [12]. about 50% lower than the maximal stimuli observed here. This is consistent with a combination of factors, such as any pre-stress induced by the patch clamp pipette [13] and some leakage associated with a less tight seal between the FluidFM tip and the cell membrane when operated with no pre-aspiration [14]. In general, a direct comparison with other cell types remains difficult, since the effects of resting membrane tension, lipid composition, and cytoskeletal mechanics on Piezo1 activation are only starting to be unraveled. As a matter of reference, HFF1 fibroblasts studied here exhibit around half the resting membrane tension compared to HEK293 cells used for many studies of Piezo1 fundamental mechanisms [12][15][16], which correlates with the 50% difference between measured critical aspiration pressures. Moreover, the linear tension regime observed in the pC,50(F) in Fig3c is expected to be valid until the indenting tip engages with other cellular organelles, as recently proposed [17]. FluidFM provides the necessary force control and sensitivity to address both small and large stimuli, and thereby study different regimes of the cellular tension machinery.

<u>SI_7:</u> Cell-wide tension changes

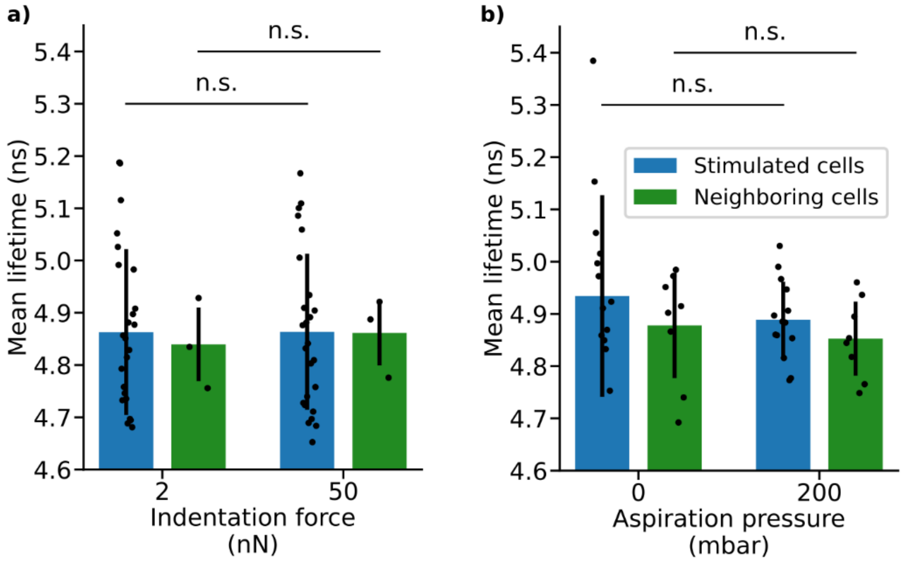

Individual and average values of fluorescence lifetime of Flipper-TR during a) indentation and b) aspiration on the outer membrane of stimulated and non-stimulated neighboring cells. Error bars correspond to one standard deviation for 25 stimulated and 3 neighboring independently measured cells for indentation, and for 14 stimulated and 8 neighboring independently measured cells for aspiration. The p-values for stimulated and neighboring cells between low and high indentation and aspiration were determined by a two-sided t-test to 0.9892, 0.7593, 0.4336 and 0.5952, respectively.

<u>SI_8:</u> Kymographs of intact cells and blebs

a)

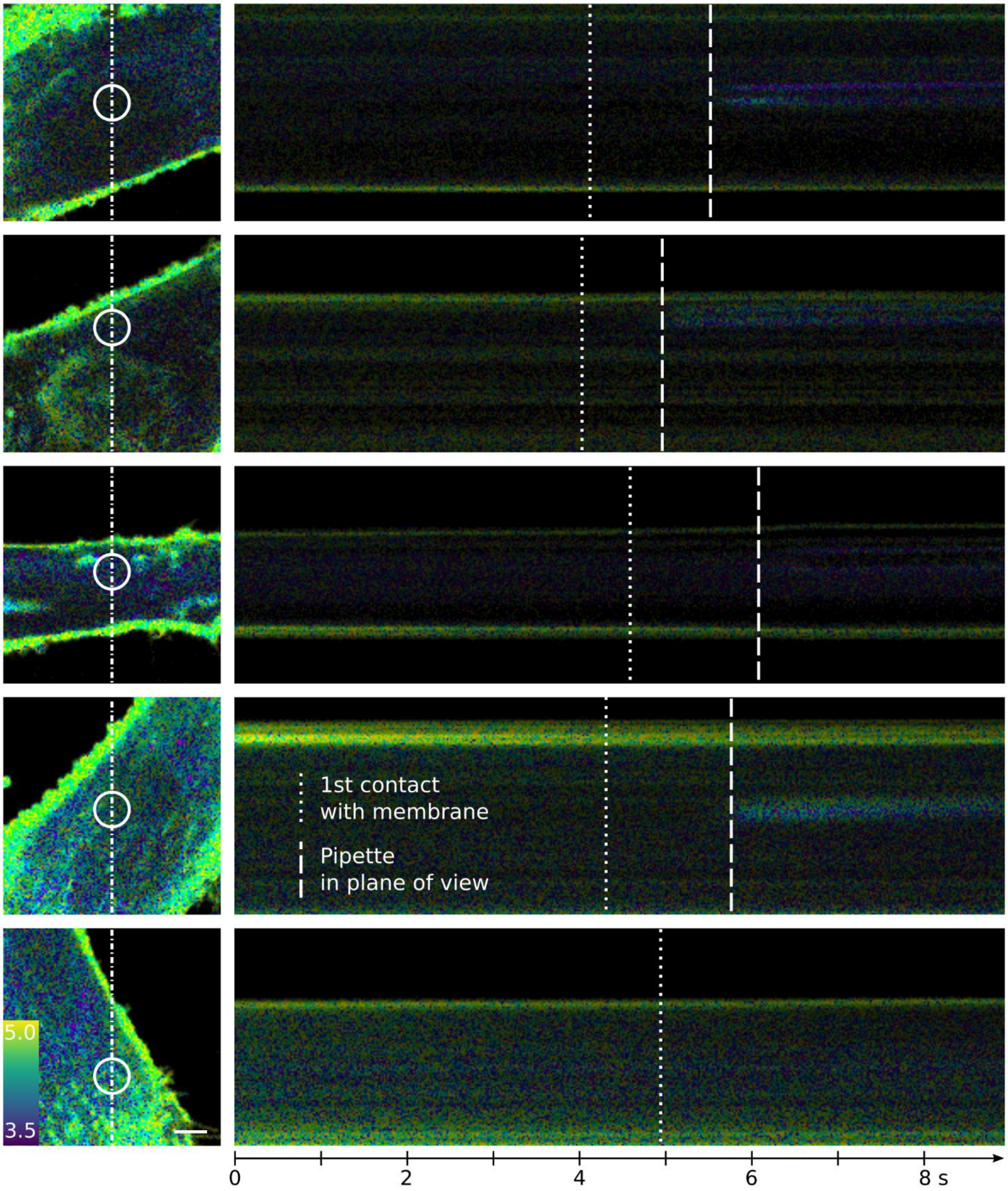

b)

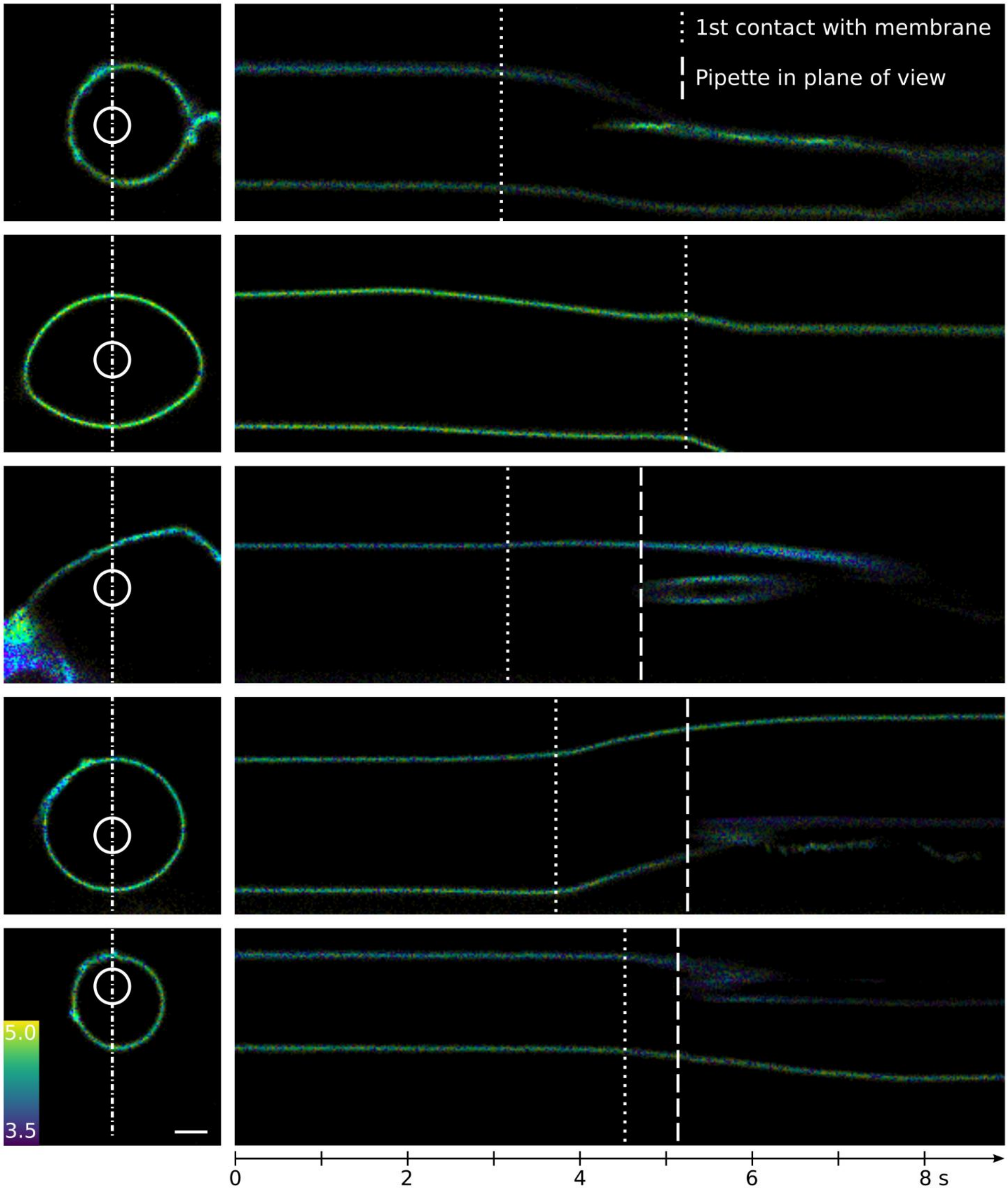

Kymographs for real-time fluorescence lifetime imaging of Flipper-TR on the cross section of five intact cells (a) and five membrane blebs (b), during indentation with 50 nN (scale bar: 2 µm, timescale: 20 ms/px). Dotted lines indicate the first contact of the FluidFM probe with the membrane, while dashed lines indicate the timepoint of the FluidFM probe entering the plane of view.

<u>SI_9:</u> Individual mechanosensitivity measurements of chemically altered cells

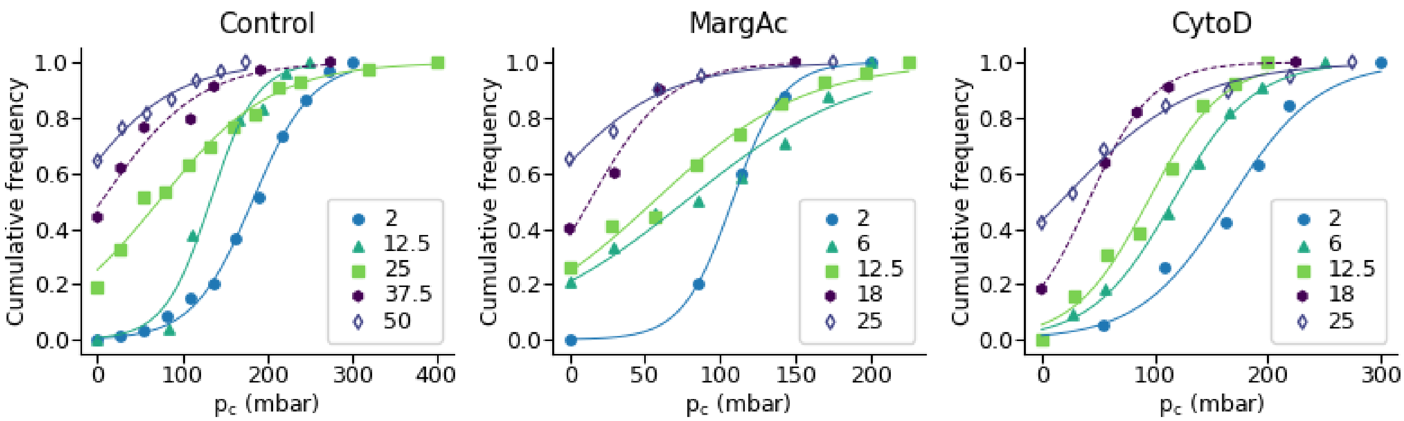

Cumulative frequency of critical aspiration pressures pC measured for each indentation force, fit with a sigmoid function to determine pC,50 as the pressure of half-maximal response, measured in 3 independent repeats with ≥10 cells per force and condition.

SI_10: Setup and Calibration of Computational Model

Cell model

The coarse-grain particle-based model of the portion of the cell was built based on the previously developed eukaryotic cell model [18]. The model was implemented using LAMMPS [19] and simulations were performed at Swiss National Supercomputer Center (CSCS). The model had 3 main components: cell membrane, cortex and cytoskeleton.

The membrane model consisted of particles connected with springs forming membrane surface triangulation. The model took into account effects of bending and in-plane shear energy, as well as viscous properties of the membrane. Global area and volume constraints were not used since only portion of the cell was modeled, however constraint of local area of elements in membrane triangulation was employed in simulations [20][21][22][23].

Specifically, the total energy of the membrane is defined as

$$V = V_S + V_b$$

where V_S is the elastic energy and V_b is the bending resistance of the membrane. The elastic energy V_S is given by

$$V_S = \sum_{i \in springs} \left[\frac{k_B T l_m}{4p} \frac{3x_i^2 - 2x_i^3}{1 - x_i} \right] + \sum_{\alpha \in triangles} \frac{1}{A_\alpha} \left[\frac{3^{3/2} k_B T l_m^3 x_0^4}{64p} \frac{4x_0^2 - 9x_0 + 6}{1 - x_0^2} \right]$$

where $k_B T$ is the energy unit, $A_\alpha$is the area of triangle α formed by three vertices in membrane surface triangulation. $x_i = l_i / l_m$, $x_0 = l_0 / l_m$, where l_i is the length of spring i, l_0 and l_m are the equilibrium spring length and maximum spring extension, p is the persistence length. The bending resistance of the membrane is modeled by

$$V_b = \sum_{\alpha,\beta\ pair} k_b [1 - \cos(\theta_{\alpha\beta} - \theta_0)]$$

where k_b is the bending constant, $\theta_{\alpha\beta}$ is the instantaneous angle between two adjacent triangles having common edge, and $\theta_0$is an equilibrium angle. The cell membrane viscoelasticity was imposed by introducing a viscous force on each spring, which has the following form

$$F_{ij}^D = -\gamma^T v_{ij} - \gamma^C (v_{ij} e_{ij}) e_{ij}$$

$$F_{ij}^R dt = \sqrt{2k_B T} \left(\sqrt{2\gamma^T} \overline{dW_{ij}^S} + \frac{\sqrt{3\gamma^C - \gamma^T} tr[dW_{ij}]}{3} \vec{1} \right) e_{ij}$$

where γ^D and γ^C are dissipative parameters, and $\overline{dW_{ij}^S}$ is the traceless symmetric part of the Wiener increments matrix.

The cytoskeleton and cortex models consisted of random network of filaments connected to each other via cross-links [18][24]. Filaments were assembled from particles connected with links. Each filament in the coarse-grained network represented a mixture of actin filaments, intermediate filaments and microtubules located inside a typical eukaryotic cell. Each cross-link was modeled by a single particle that could link to two filaments. The harmonic potentials were employed to describe the extension and bending of filaments and cross-links

$$E_{bond} = \kappa_{ext}(r - r_0)^2, E_{angle} = \kappa_{bend}(\theta - \theta_0)^2$$

where $\kappa_{ext}$is extensional stiffness, $r_0$is equilibrium length, $\kappa_{bend}$is bending stiffness, and $\theta_0$is equilibrium angle. In order to reduce the total number of model parameters, the cortex was modeled using the same type of filaments and cross-links within the cytoskeleton network by specifying higher density of filaments and cross-links in the cortex region with preferred orientation of the filaments aligned along the membrane.

After initially placing filaments and cross-links in the periodic rectangular domain at random, simulations were performed in which cross-links could bind filaments if the distance between cross-link particle and filament particle was less than

certain threshold, the cross-links could also unbind from the filaments in a force-dependent manner. The network was allowed to evolve until reaching steady dynamic state. At this point, rectangular portion of the network with cortex was cut out and placed in simulation box together with the membrane model [18][24]. The membrane was then connected to the cortex region of the cytoskeleton network in simulations, where the links were spontaneously formed if the distance between cortex filament and membrane particles was smaller than a threshold, stretched links were broken in the force-dependent manner. The simulations were run until the number of links between the cortex and the membrane converged to a steady value [18][24].

Typical model obtained following the procedure described above is shown in Fig5a. A portion of a cell, 15 × 15 μm^2 and 5 μm in thickness was simulated. The membrane model consisted of 16000 particles. The average length of the membrane links was equal to 0.125 μm. The membrane particles were connected to the cell cortex filaments using about 5000 links of 0.125 μm length. Each filament in the cortex and cytoskeleton models had length equal to 1 μm and was modeled using 9 particles. The cytoskeleton network consisted of 40000 filaments with 10000 filaments located in the cortex region. The width of the cortex region adjacent to the membrane was equal to 0.2 μm in the model. Total of 80000 cross-link springs were used to connect filaments in the cytoskeleton and cortex models. The equilibrium length of the cross-link links connecting filaments was 0.0625 μm. It was assumed that the cell was firmly attached to the substrate in experiments, which was modeled by freezing the lower 0.25 μm portion of the network. Similarly, 0.25 μm wide portion of the network was frozen at the sides of the simulation domain, in order to take into account the effect of cell beyond the simulations domain. Detailed description of the cell model equations and parameters can be found in [18][24].

Dissipative particle dynamics

Fluid inside and outside of the cell was present in all simulations and modeled using particles. A coarse-grained Molecular Dynamics (MD) method, Dissipative Particle Dynamics (DPD), was chosen as a simulation framework, providing a unifying way to describe cell, fluid, and their interactions [20][25][26][27]. The fluid, membrane, cytoskeleton and cortex viscous properties were controlled by DPD dissipative and random forces acting on particles.

In DPD method, the system is modeled by collection of particles, with each particle representing a center of mass of cluster of atoms or molecules. The time evolution of positions and velocities of the DPD particles in the system are described by Newton's equations of motion,

$$\frac{dr_i}{dt} = v_i \, , \frac{dv_i}{dt} = f_i$$

where r_i and v_i are position and velocity of particle i. The force f_i which acts on particle i is expressed by three additive parts,

$$f_i = \sum_{i \neq j} f_{ij}^C + f_{ij}^D + f_{ij}^R$$

which are non-zero within a cutoff radius of R_C. The conservative force f_{ij}^C is a soft repulsion force, acting along the vector between particles i and j with a parameter a_{ij} defining the maximum repulsion between the two particles,

$$f_{ij}^C = \begin{cases} a_{ij}\left(1 - \frac{r_{ij}}{R_C}\right)\widehat{r_{ij}}, & r_{ij} < R_C \\ 0, & r_{ij} \geq R_C \end{cases}$$

where $r_{ij} = |r_i - r_j|$and $\widehat{r_{ij}} = (r_i - r_j)/r_{ij}$. The dissipative force, f_{ij}^D, and the random force, f_{ij}^R are expressed as

$$f_{ij}^D = -\gamma w^D(r_{ij})(\widehat{r_{ij}} v_{ij})\widehat{r_{ij}}, \qquad f_{ij}^R = \sigma w^R(r_{ij})\theta_{ij}\widehat{r_{ij}}$$

where$v_{ij} = v_i - v_j$, and θ_{ij}stands for a random variable with zero mean and unit variance. These 2 forces form the DPD thermostat and are related by the fluctuation-dissipation theorem [25]. Additionally, we used a generalized weighting function:

$$w^R(r_{ij}) = \left(1 - \frac{r_{ij}}{R_C}\right)^{0.25}$$

The soft conservative force employed in DPD allows to use much larger time integration step in comparison to MD, thus making DPD efficient mesoscale simulation method, often employed in simulations of soft matter. For all the simulations in this work, DPD parameters were taken from reference [18].

Pipette and Membrane Interactions

The micropipette was modeled using level-sets with solid wall boundary conditions imposed on its surface as in [18][23][24][28][29]. Preliminary simulations of micropipette aspiration revealed that adhesion of the membrane to pipette played an important role in distribution of membrane tension during indentation and aspiration. Without adhesion, large increase of membrane tension was observed in simulations in the area of contact between micropipette and membrane. This was in contrast to experimental measurements, where membrane tension increased similarly at the rim and inside the pipette. Adding adhesion of the membrane particles to the surface of the pipette at contact provided better agreement with experiment, and therefore was used in all simulations.

Calibration of the Cell Model

The cytoskeleton of the eukaryotic cell is very complex, consisting of many filaments of different types with various cross-linking proteins. Explicitly taking into account this complexity in computation model would be impractical considering the time and length scales of the experiments. Therefore, no attempt was made to directly relate parameters of the model to the molecular parameters of various cell components. Instead, the model parameters were chosen to match cell response in experiments, following the procedure described in [18][24].

Specifically, it was observed in control experiments with adherent fibroblasts, that with increasing indentation depth, less aspiration pressure was necessary to evoke a mechanosensitive calcium response as shown in Fig3c. Increase of indentation depths from 0.643 to 1.588 and 1.985 µm, resulted in decrease of critical aspiration pressure by 20 and 44 percent, respectively. In addition, the length of membrane insertion into pipette during indentation were measured to be less than 0.5 and 1 µm at indentation force of 2 and 25 nN, respectively.

To define the values of cell model parameters, series of simulations of micropipette indentation and aspiration were performed, allowing to tune model parameters until the desired experimental measurements were matched. For each set of model parameters, indentation simulations were first performed with prescribed indentation depth of 0.643, 1.588 and 1.985 µm. The length of the membrane insertion in the pipette was measured and ensured to be within the range of those observed experimentally. For each indentation depth, simulations of micropipette aspiration were performed, in which dependence of the membrane tension was recorded as a function of the aspiration pressure. It was assumed that in experiments the critical membrane tension evoking cell response was the same in all control experimental conditions. With this assumption, a set of model parameters was identified that gave the same critical membrane tension at aspiration pressures decreasing by 20 and 44% at indentation depths of 1.588 and 1.985 µm, comparing to aspiration pressure at 0.643 µm indentation. The critical membrane tension was calculated as an average over highest 10% of the membrane tension inside the pipette. It was not possible to fit model results to experimental data when critical pressure in simulation was calculated as an average over the entire membrane inside the pipette, suggesting that the cell response is triggered by local increase of membrane tension (and the number of activated Piezo1 channels) during micropipette aspiration.

In simulations where variation of cell stiffness was considered, it was achieved by changing of extensional stiffness κ_{ext} and bending stiffness κ_{bend} coefficients of filaments and crosslinks of the cytoskeleton network model, while keeping network structure unchanged. Variation of resting membrane tension was achieved by changing equilibrium area of elements in surface triangulation of the membrane model.

Supplementary References

1. A. Colom, E. Derivery, S. Soleimanpour, C. Tomba, M. D. Molin, N. Sakai, M. González-Gaitán, S. Matile, & A. Roux, A fluorescent membrane tension probe. *Nature Chemistry*, **10** (2018) 1118–1125. https://doi.org/10.1038/s41557-018-0127-3.

2. M. Sergides, L. Perego, T. Galgani, C. Arbore, F. S. Pavone, & M. Capitanio, Probing mechanotransduction in living cells by optical tweezers and FRET-based molecular force microscopy. *European Physical Journal Plus*, **136** (2021) 316. https://doi.org/10.1140/epjp/s13360-021-01273-7.

3. C. Arbore, L. Perego, M. Sergides, & M. Capitanio, Probing force in living cells with optical tweezers: from single-molecule mechanics to cell mechanotransduction. *Biophysical Reviews*, **11** (2019) 765–782. https://doi.org/10.1007/s12551-019-00599-y.

4. M. Capitanio, G. Romano, R. Ballerini, M. Giuntini, F. S. Pavone, D. Dunlap, & L. Finzi, Calibration of optical tweezers with differential interference contrast signals. *Review of Scientific Instruments*, **73** (2002) 1687. https://doi.org/10.1063/1.1460929.

5. E. Sitarska & A. Diz-Muñoz, Pay attention to membrane tension: Mechanobiology of the cell surface. *Current Opinion in Cell Biology*, **66** (2020) 11–18. https://doi.org/10.1016/j.ceb.2020.04.001.

6. W. E. Brownell, F. Qian, & B. Anvari, Cell membrane tethers generate mechanical force in response to electrical stimulation. *Biophysical Journal*, **99** (2010) 845–852. https://doi.org/10.1016/j.bpj.2010.05.025.

7. W. Yu, P. T. C. So, T. French, & E. Gratton, Fluorescence generalized polarization of cell membranes: A two-photon scanning microscopy approach. *Biophysical Journal*, **70** (1996) 626–636. https://doi.org/10.1016/S0006-3495(96)79646-7.

8. B. M. Gaub & D. J. Müller, Mechanical Stimulation of Piezo1 Receptors Depends on Extracellular Matrix Proteins and Directionality of Force. *Nano Letters*, **17** (2017) 2064–2072. https://doi.org/10.1021/acs.nanolett.7b00177.

9. O. Guillaume-Gentil, C. G. Gäbelein, S. Schmieder, V. Martinez, T. Zambelli, M. Künzler, & J. A. Vorholt, Injection into and extraction from single fungal cells. *Communications Biology*, **5** (2022) 1–10. https://doi.org/10.1038/s42003-022-03127-z.

10. P. Dörig, D. Ossola, A. M. Truong, M. Graf, F. Stauffer, J. Vörös, & T. Zambelli, Exchangeable colloidal AFM probes for the quantification of irreversible and long-term interactions. *Biophysical Journal*, **105** (2013) 463–472. https://doi.org/10.1016/j.bpj.2013.06.002.

11. E. Potthoff, D. Ossola, T. Zambelli, & J. A. Vorholt, Bacterial adhesion force quantification by fluidic force microscopy. *Nanoscale*, **7** (2015) 4070–4079. https://doi.org/10.1039/c4nr06495j.

12. A. H. Lewis & J. Grandl, Mechanical sensitivity of Piezo1 ion channels can be tuned by cellular membrane tension. *eLife*, **4** (2015) e12088. https://doi.org/10.7554/eLife.12088.

13. O. P. Hamill & D. W. McBride, INDUCED MEMBRANE HYPO/HYPER-MECHANOSENSITIVITY:A Limitation of Patch-Clamp Recording. *Annual Review of Physiology*, **59** (1997) 621–631. https://doi.org/10.1146/annurev.physiol.59.1.621.

14. D. Ossola, M. Y. Amarouch, P. Behr, J. Vörös, H. Abriel, & T. Zambelli, Force-controlled patch clamp of beating cardiac cells. *Nano Letters*, **15** (2015) 1743–1750. https://doi.org/10.1021/nl504438z.

15. C. D. Cox, C. Bae, L. Ziegler, S. Hartley, V. Nikolova-Krstevski, P. R. Rohde, C. A. Ng, F. Sachs, P. A. Gottlieb, & B. Martinac, Removal of the mechanoprotective influence of the cytoskeleton reveals PIEZO1 is gated by bilayer tension. *Nature Communications*, **7** (2016) 10366. https://doi.org/10.1038/ncomms10366.

16. P. Ridone, E. Pandzic, M. Vassalli, C. D. Cox, A. Macmillan, P. A. Gottlieb, & B. Martinac, Disruption of membrane cholesterol organization impairs the activity of PIEZO1 channel clusters. *Journal of General Physiology*, **152** (2020) e201912515. https://doi.org/10.1085/jgp.201912515.

17. H. De Belly, S. Yan, H. Borja da Rocha, S. Ichbiah, J. P. Town, P. J. Zager, D. C. Estrada, K. Meyer, H. Turlier, C. Bustamante, & O. D. Weiner, Cell protrusions and contractions generate long-range membrane tension propagation. *Cell*, **186** (2023) 3049-3061.e15. https://doi.org/10.1016/j.cell.2023.05.014.

18. K. Lykov, Y. Nematbakhsh, M. Shang, C. T. Lim, & I. V. Pivkin, Probing eukaryotic cell mechanics via mesoscopic simulations. *PLoS Computational Biology*, **13** (2017) e1005726. https://doi.org/10.1371/journal.pcbi.1005726.

19. S. Plimpton, Fast parallel algorithms for short-range molecular dynamics. *Journal of Computational Physics*, **117** (1995) 1–19. https://doi.org/10.1006/jcph.1995.1039.

20. I. V. Pivkin & G. E. Karniadakis, Accurate coarse-grained modeling of red blood cells. *Physical Review Letters*, **101** (2008). https://doi.org/10.1103/PhysRevLett.101.118105.

21. H. Bow, I. V. Pivkin, M. Diez-Silva, S. J. Goldfless, M. Dao, J. C. Niles, S. Suresh, & J. Han, A microfabricated deformability-based flow cytometer with application to malaria. *Lab Chip* (Royal Society of Chemistry, 2011), pp. 1065–1073. https://doi.org/10.1039/c0lc00472c.

22. I. V. Pivkin, Z. Peng, G. E. Karniadakis, P. A. Buffet, M. Dao, & S. Suresh, Biomechanics of red blood cells in human spleen and consequences for physiology and disease. *Proceedings of the National Academy of Sciences of the United States of America*, **113** (2016) 7804–7809. https://doi.org/10.1073/pnas.1606751113.

23. D. Rossinelli, Y. H. Tang, K. Lykov, D. Alexeev, M. Bernaschi, P. Hadjidoukas, M. Bisson, W. Joubert, C. Conti, G. Karniadakis, M. Fatica, I. Pivkin, & P. Koumoutsakos, The in-silico lab-on-a-chip: Petascale and high-throughput simulations of microfluidics at cell resolution. *Int. Conf. High Perform. Comput. Networking, Storage Anal. SC* (IEEE Computer Society, 2015). https://doi.org/10.1145/2807591.2807677.

24. K. Lykov, Cell mechanics in flow : algorithms and applications Kirill Lykov. *Thesis*, (2017).

25. R. D. Groot & P. B. Warren, Dissipative particle dynamics: Bridging the gap between atomistic and mesoscopic simulation. *Journal of Chemical Physics*, **107** (1997) 4423–4435. https://doi.org/10.1063/1.474784.

26. I. V. Pivkin, B. Caswell, & G. E. Karniadakis, Dissipative particle dynamics. *Reviews in Computational Chemistry*, **27** (2010) 85–110. https://doi.org/10.1002/9780470890905.ch2.

27. Z. Li, X. Bian, X. Li, M. Deng, Y. Tang, B. Caswell, & G. E. Karniadakis, *Dissipative Particle Dynamics : Foundation , Evolution , Implementation , and Applications*. https://doi.org/10.1007/978-3-319-60282-0.

28. I. V. Pivkin & G. E. Karniadakis, Controlling density fluctuations in wall-bounded dissipative particle dynamics systems. *Physical Review Letters*, **96** (2006) 206001. https://doi.org/10.1103/PhysRevLett.96.206001.

29. I. V. Pivkin & G. E. Karniadakis, A new method to impose no-slip boundary conditions in dissipative particle dynamics. *Journal of Computational Physics*, **207** (2005) 114–128. https://doi.org/10.1016/j.jcp.2005.01.006.
