# Supplementary material for: Dissecting cell membrane tension dynamics and its effect on Piezo1-mediated cellular mechanosensitivity using force-controlled nanopipettes
Source: Nat Methods. 2024 May 27;21(6):1063–73. doi: 10.1038/s41592-024-02277-8 (PMC11166569; doi:10.1038/s41592-024-02277-8)
Supplement: Supplementary file 2 — Reporting Summary [file 41592_2024_2277_MOESM2_ESM.pdf]

nature portfolio

Corresponding author(s): Ines Lüchtefeld, Massimo Vassalli

Last updated by author(s): Feb 12, 2024

Reporting Summary

Nature Portfolio wishes to improve the reproducibility of the work that we publish. This form provides structure for consistency and transparency in reporting. For further information on Nature Portfolio policies, see our Editorial Policies and the Editorial Policy Checklist.

Statistics

For all statistical analyses, confirm that the following items are present in the figure legend, table legend, main text, or Methods section.

n/a	Confirmed	
☐	☒	The exact sample size (*n*) for each experimental group/condition, given as a discrete number and unit of measurement
☐	☒	A statement on whether measurements were taken from distinct samples or whether the same sample was measured repeatedly
☐	☒	The statistical test(s) used AND whether they are one- or two-sided<br>*Only common tests should be described solely by name; describe more complex techniques in the Methods section.*
☒	☐	A description of all covariates tested
☒	☐	A description of any assumptions or corrections, such as tests of normality and adjustment for multiple comparisons
☐	☒	A full description of the statistical parameters including central tendency (e.g. means) or other basic estimates (e.g. regression coefficient) AND variation (e.g. standard deviation) or associated estimates of uncertainty (e.g. confidence intervals)
☐	☒	For null hypothesis testing, the test statistic (e.g. *F*, *t*, *r*) with confidence intervals, effect sizes, degrees of freedom and *P* value noted<br>*Give P values as exact values whenever suitable.*
☒	☐	For Bayesian analysis, information on the choice of priors and Markov chain Monte Carlo settings
☒	☐	For hierarchical and complex designs, identification of the appropriate level for tests and full reporting of outcomes
☒	☐	Estimates of effect sizes (e.g. Cohen's *d*, Pearson's *r*), indicating how they were calculated

Our web collection on statistics for biologists contains articles on many of the points above.

Software and code

Policy information about availability of computer code

Data collection	Calcium imaging data were collected with the open-source micromanager microscopy software. FLIM data were collected with the commercial LAS X software (Leica). AFM data were collected with a custom LabView script.
Data analysis	The commercial LAS X software (Leica) was used to analyse FLIM lifetimes. Python 3.6 was used to process calcium videos (using the python package Noise2Void) and FLIM images.

For manuscripts utilizing custom algorithms or software that are central to the research but not yet described in published literature, software must be made available to editors and reviewers. We strongly encourage code deposition in a community repository (e.g. GitHub). See the Nature Portfolio guidelines for submitting code & software for further information.

Data

Policy information about availability of data

All manuscripts must include a data availability statement. This statement should provide the following information, where applicable:

- Accession codes, unique identifiers, or web links for publicly available datasets
- A description of any restrictions on data availability
- For clinical datasets or third party data, please ensure that the statement adheres to our policy

Full data sets are available in the data repository published here: https://doi.org/10.3929/ethz-b-000659903. The data repository includes complete image data sets for the step-wise and real-time measurement of Flipper-TR. For the calcium imaging data, exemplary image files are included, due to the large size of the full data

set. The full data set for calcium imaging is available from the corresponding authors upon request and a file including all extracted datapoints are included in the repository.

Human research participants

Policy information about studies involving human research participants and Sex and Gender in Research.

Reporting on sex and gender	N/A
Population characteristics	N/A
Recruitment	N/A
Ethics oversight	N/A

Note that full information on the approval of the study protocol must also be provided in the manuscript.

Field-specific reporting

Please select the one below that is the best fit for your research. If you are not sure, read the appropriate sections before making your selection.

☒ Life sciences ☐ Behavioural & social sciences ☐ Ecological, evolutionary & environmental sciences

For a reference copy of the document with all sections, see nature.com/documents/nr-reporting-summary-flat.pdf

Life sciences study design

All studies must disclose on these points even when the disclosure is negative.

Sample size	No statistical method was used to predetermine sample size since distributions were unknown for this new data type, but a minimum of three independent replicates were performed for all experiments. For mechanical stimulation and calcium imaging, 3x 20 cells were sufficient to show significant differences between conditions. For FLIM imaging, 4-5 cells were sufficient to show clear trends.
Data exclusions	In figure 3b and 4a non-responding cells and ruptured cells were excluded since they do not yield a critical aspiration pressure. These exclusion criteria were pre-established.
Replication	All experiments were performed on at least 3 independent sets of cells, and trends were consistent between sets.
Randomization	Samples were allocated randomly to experimental groups.
Blinding	Blinding was not performed, since the workflow is automated and an influence of the operator is minimal.

Reporting for specific materials, systems and methods

We require information from authors about some types of materials, experimental systems and methods used in many studies. Here, indicate whether each material, system or method listed is relevant to your study. If you are not sure if a list item applies to your research, read the appropriate section before selecting a response.

Materials & experimental systems

n/a	Involved in the study	
☒	☐	Antibodies
☐	☒	Eukaryotic cell lines
☒	☐	Palaeontology and archaeology
☒	☐	Animals and other organisms
☒	☐	Clinical data
☒	☐	Dual use research of concern

Methods

n/a	Involved in the study	
☒	☐	ChIP-seq
☒	☐	Flow cytometry
☒	☐	MRI-based neuroimaging

Eukaryotic cell lines

Policy information about cell lines and Sex and Gender in Research

Cell line source(s)	Cell line HFF-1 obtained from ATCC

Authentication	Authenticated by ATCC (by STR profiling and morphology), used directly from supplier
Mycoplasma contamination	No mycoplasma contamination was detected, as tested by ATCC.
Commonly misidentified lines (See ICLAC register)	None
